# An update about beneficial effects of medicinal plants in aquaculture: A review

**DOI:** 10.17221/96/2023-VETMED

**Published:** 2023-12-26

**Authors:** Faranak Dadras, Josef Velisek, Eliska Zuskova

**Affiliations:** Faculty of Fisheries and Protection of Waters, South Bohemian Research Center of Aquaculture and Biodiversity of Hydrocenoses, Research Institute of Fish Culture and Hydrobiology, University of South Bohemia in Ceske Budejovice, Ceske Budejovice, Czech Republic

**Keywords:** disease, fish, growth, immunity, medicinal herb

## Abstract

Aquaculture is an essential and growing component of agricultural and global ecosystems worldwide. Aquaculture provides more than 25% of the total aquatic food consumption by humans. The development of the aquaculture industry should be followed in successive industrial years, and therefore it is necessary to pay attention to the management and type of farming system that is compatible with the environment. The use of antibiotics for disease control has been criticised for their negative effects, including the emergence of antibiotic-resistant bacteria, the suppression of the immune system and the environment, and the accumulation of residue in aquatic tissues. The use of these products reduces the need for treatments, enhances the effect of vaccines, and, in turn, improves production indicators. Medicinal plants have increasingly been used in recent years as a disease control strategy in aquaculture, boosting the immune system of aquatic animals and helping to develop strong resistance to a wide range of pathogens. Therefore, this review aims to provide an overview of the recent evidence on the beneficial use of medicinal plants to promote growth and strengthen the immune system in farmed aquatic animals.

## INTRODUCTION

Aquaculture has been forecasted to increase by 62% between 2010 and 2030 to supply the growing fish and seafood demand derived from a steadily growing population, providing over two-thirds of the total fish and shellfish consumed worldwide (Bank 2013; Sofia 2018). On the other hand, in 2020, the total amount of globally farmed fish amounted to 82.1 million tonnes, confirming aquaculture remains one of the significant contributors to animal protein sources (Stankus 2021). Despite the considerable role of aquaculture, the sector faces many challenges that prevent its expansion. The high culture density has led to environmental pollution and the emergence of diseases (Lafferty et al. 2015) due to stressed and immuno-compromised animals (Bondad-Reantaso et al. 2005; Pulkkinen et al. 2010). Other factors, such as storms, droughts, and high temperatures, adversely affect the water quality and may compromise the fish’s immune system and the health of aquatic animals (Dubey et al. 2017; Abdel-Tawwab et al. 2019).

Aquatic animal diseases are among the key limiting factors for aquaculture development (Stentiford et al. 2017), which could lead to the partial or complete loss of growth and production and result in considerable economic losses, estimated at over 9.5 billion USD per year (Shinn et al. 2015; Ramesh and Souissi 2018). Therefore, antibiotics and other veterinary drugs are commonly used in aquaculture to treat disease outbreaks as well as to prevent and mitigate the economic losses resulting from sanitary shortcomings (Rico et al. 2013; Cabello et al. 2016; Miranda et al. 2018). The potential risks of using antibiotics and the associated global health threats due to the selection and emergence of antibiotic-resistant bacteria in aquaculture have been extensively studied (Cabello et al. 2016; Chen et al. 2018). Recently, in response to the risk of developing resistant genes that could potentially change the human pathogen, the use of antibiotics has been restricted in aquaculture (Binh et al. 2018; van Wyk and Prinsloo 2020). Several alternative strategies, including vaccination, probiotics, herbal plants, and immunostimulants, have been proposed to prevent disease outbreaks and limit the use of veterinary drugs in aquaculture (Liu et al. 2014; Reverter et al. 2014; Reverter et al. 2017). Vaccination is a precise technique that requires a clear disease diagnosis (Brudeseth et al. 2013) as well as the complicated development of multiple-strain vaccines (Sakai 1999; Pasnik et al. 2005). Furthermore, it is too expensive for widespread use among small-scale fish farmers (Pridgeon and Klesius 2012).

As one of the best alternatives, medicinal plants have been strongly recommended. Medicinal plants contain specific active substances modulating biological functions, such as growth promotion (Amin et al. 2019; Gharaei et al. 2020), anti-stress effects (Abd El-Gawad et al. 2020), appetite stimulation (Mo et al. 2016), immunostimulation (Rufchaei et al. 2017), disease resistance (Liu et al. 2010a; van Wyk and Prinsloo 2020), and antimicrobial activities (Ardo et al. 2008; Beltran et al. 2018). To ensure sustainable aquaculture, medicinal plants seem to be cheaper and more sustainable alternatives to chemotherapy. This supports the increasing number of publications on conceptualising medicinal plant applications. The present review purposefully discussed the adoption of naturally available plants in the form of roots, leaves, flowers, and in a processed form, including active compounds, extracts, crude forms, and mixed with different promising effects in aquaculture.

### Application of medicinal plants in aquaculture

Medicinal plants contain antioxidant compounds with a high affinity to trap free radicals inhibiting the normal functioning of cells (Moreno et al. 2020). Many antimicrobial substances in these plants make them an appropriate candidate to fight a wide range of pathogenic microbes (Jafarzadeh et al. 2020). Large bioactive compounds, including steroids, proteins, tannins, saponins, terpenoids, and alkaloids, are found in different plants. These compounds have shown strong resistance to a wide range of bacteria pathogens (*Salmonella typhi*, *Bordetella pertussis*, *Corynebacterium parvum*, *Klebsiella pneumoniae*, *Mycobacterium* and *Escherichia coli*), fungi (*Aspergillus flavus*, *Aspergillus fumigatus*, *Fusarium solani* and *Pseudomonas aeruginosa*) and viruses (*retrovirus*, *simian-virus*) (Ma et al. 2019).

Medicinal plants have been used in chemotherapeutics and as feed additives (Reverter et al. 2014; Garg et al. 2020). For a long time, they have been used by rural fish farmers (Reverter et al. 2017); in 46% of the surveyed fish farmers (Caruso et al. 2013). Medicinal plants, as sustainably used candidates, are easily accessible and readily available to be applied in intensive farming in aquaculture to ensure the improved productivity and health status of aquatic organisms (Cawthorn and Hoffman 2015). Medicinal plants can be administered in different ways, either as a whole plant or in parts (leaf, root, seed, fruit) and can either be applied fresh or as prepared herbal extracts with different solvents (water, methanol, chloroform, ethyl acetate) (Van Hai 2015). The biological activity and chemical composition of plants and extracts can vary greatly depending on the part used and type of extract, thus, knowledge of the plant’s bioactive compounds is required.

For instance, some herbs, such as moringa (*Moringa oleifera*), can be used as a protein source or partial protein replacement due to its high protein content, i.e., 27.51% in the crude protein of the leaves (Oduro et al. 2008). Selection of a suitable dosage of a medicinal herb is crucial to obtain the desired effects since inappropriate doses can display toxic effects (Sambasivam et al. 2003; Kavitha et al. 2012). The high activation of the immune response without harmful and toxic effects on animals can be induced by a proper dose of the target material (Bulfon et al. 2013). Some parts of the produced chemical compounds during regular metabolic activities of the plants, such as phytochemicals, comprise a diverse group of natural products. They may be nutritionally essential, but many of them, such as phytate, lectin, and trypsin inhibitors, have no nutritional value and, having antinutritional properties, can decrease the feed conversion efficiency and growth rate (Makkar et al. 2007; Hashemi and Davoodi 2011). A study on the toxic effect of Indian almonds (*Terminalia catappa*), leaf extracts at different concentrations (700, 800, 900, 1 000, and 1 100 mg/l) on the Nile tilapia (*Oreochromis niloticus*) juveniles under static toxicity exposure (96 h exposure) showed the 96hLC50 value was 900 mg/l (Yunus et al. 2019).

A diet supplemented with a 1% ethanol katuk extract (*Sauropus androgynus*) showed enhanced growth and improved food utilisation in the orange-spotted grouper (*Epinephelus coioides*), whereas 2.5% and 5% of the katuk extract presented lower growth levels (Santoso et al. 2013). Another critical factor in a medicinal plant application is the treatment length. Farmed barramundi (*Lates calcarifer*) fed an enriched diet of garlic (*Allium sativum*) for 30 days showed a 70% decreased *Neobenedenia* sp. infection compared to the control and the group fed garlic for 10 days (Militz et al. 2013).

Medicinal plants can be administered to fish by injection (intramuscular and intraperitoneal), oral administration, or immersion (Wu et al. 2010; Ji et al. 2012; Santoso et al. 2013). The intraperitoneal injection is considered the most rapid and efficient method of administration; however, it is expensive, laborious, and stressful for the fish (Anderson 1992; Yoshida et al. 1995); on the other hand, baths are extensively used for the treatment of ectoparasites (Whittington 2012; Forwood et al. 2013), but this method is also expensive and laborious and involves the release of exogenous molecules in the marine environment (Umeda et al. 2006). Zebrafish (*Danio rerio*) received an injection of a coumarin derivative (10 μl/fish) for 14 days and showed improved resistance against spring viraemia of carp virus (SVCV) (Shen et al. 2018). An oral administration of berberine hydrochloride (30–50 mg/kg) in Prussian carp (*Carassius gibelio*) for 15 days showed antivirus activity against herpesvirus 2 (CyHV-2) (Su et al. 2021).

Tilapia were injected with a hot-water extract of Chinese mahogany (*Toona sinensis*) (with a 2 or 4 mg/ml solution) at doses of 4 or 8 mg/g and had significantly increased respiratory burst, phagocytic activity, and lysozyme activity towards *Aeromonas hydrophila* by 1 and 2 days post-injection (Wu et al. 2010). An improved survival rate and lysozyme activity were reported in rainbow trout (*Oncorhynchus mykiss*) orally administered with a Greek juniper extract at 8 mg/kg dose for 14 days (Bilen et al. 2021). The intraperitoneal injection, oral administration, and diffusion of a 0.1 ml extract of green chiretta (*Andrographis paniculata*) in Indian major carp (*Labeo rohita*) improved the non-specific immune system against *Aeromonas hydrophila* via enhancing the total erythrocyte count and normal haemoglobin levels (Palanikani et al. 2020).

### Biological activity of medicinal plants in fish

The main target organs of fish influenced by medicinal plants are the thymus, spleen, kidney, and guts, which promote immune system development. Medicinal plants can directly improve the antibody production and the specific immune response. Many medicinal plants can promote the production of cytokines that mediate the specific/non-specific immunity, including interleukin, interferon, and the tumour necrosis factor (Tadese et al. 2021).

Medicinal plants enhance immune parameters (Dugenci et al. 2003; Yuan et al. 2007). The biological activity of medicinal plants is attributed to their secondary metabolites (SMs), such as essential oils, saponins, phenolics, tannins, alkaloids, polypeptides, and polysaccharides (Hoseinifar et al. 2020b). The SMs play a key role in stress mediation, antioxidant activity, and immunopotentiation by modulating the recognition, binding, catalytic activity, and turnover of proteins and DNA (Chong et al. 2020). Several *in vitro* and *in vivo* studies have demonstrated the beneficial effects of medicinal plants against a wide range of marine pathogens (bacteria, viruses, fungi, and ectoparasites) (Direkbusarakom et al. 1996; Chitmanat et al. 2005; Ji et al. 2012; Su et al. 2021).

#### MEDICINAL PLANTS AS GROWTH PROMOTERS

As summarised in see Electronic Supplementary Material (ESM) Table S1, medicinal plants can stimulate the appetite and promote weight gain as they enhance the digestive enzyme activity (Van Hai 2015). For instance, some herbs, such as the sissoo spinach (*Alternathera sessilis*), false daisy (*Eclipta alba*), and veldt grape (*Cissus quadrangularis*) used as appetisers, improved the activities of protease, amylase, and lipase of freshwater prawns (Radhakrishnan et al. 2014). Adding a wormwood (*Artemisia annua)* extract had a growth-promoting effect on rainbow trout (Koshinski 2018) and carp (Sarhadi et al. 2020). Similar results have been reported by He et al. (2022) in largemouth black bass (*Micropterus salmoides*).

The growth rate of freshwater prawns (*Macro-brachium rosenbergii*) has been improved using a diet supplemented with an emodin extract (Liu et al. 2010b). Greasy groupers (*Epinephelus tauvina*) fed a diet supplemented with a mixture of methanolic herb extracts of Bermuda grass (*Cynodon dactylon*), Indian long pepper (*Piper longum*), stonebreaker (*Phyllanthus niruri*), Tridax daisy (*Tridax procumbens*), and ginger (*Zingiber officinalis*) showed enhanced weight gain (41%) (Punitha et al. 2008).

The administration of numerous plant extracts to cultured fish induces appetite and increases weight gain (Raja Rajeswari et al. 2012; Hoseinifar et al. 2020c; Mohammadi et al. 2020). Several studies confirmed the promising effect of medicinal plants on the growth performance of fish species, including the administration of ferula (*Ferula asafoetida*) powder in common carp (*Cyprinus carpio*) (Safari et al. 2019); prickly chaff flower (*Achyranthes aspera*) (0.5%) in rohu (*Labeo rohita*) fry (Sharma et al. 2019); onion (*Allium cepa*) powder (10 g/kg) in white carp (*Cirrhinus mrigala*) fingerlings (Sikotariya 2019); chaff flower (*Achyranthes aspera*) (0.5%) in rohu (*Labeo rohita*) (Singh et al. 2019); dandelion (*Taraxacum officinale*) extract (0.8%) in common carp (Sirakov et al. 2019); ginger (*Zingiber officinale*) (0.8%) in rohu (Labeo rohita) fingerlings (Sukumaran et al. 2016); climbing Senecio (*Senecio scandens* buch-ham) extracts (0.05–0.1%) in hybrid grouper (*Epinephelus lanceolatus*♂  × *Epinephelus fuscoguttatus*♀) (Sun et al. 2020); fluted pumpkin (*Telfairia occidentalis*) (1%) in African sharp-tooth catfish (*Clarias gariepinus*) (Ta et al. 2019); dandelion extract (1 g/kg) in golden pompano (*Trachinotus ovatus*) (Tan and Sun 2020); wolfberry (*Lycium barbarum*) extract (0.5–2%) in hybrid grouper (*Epinephelus lanceolatus*♂ × *E. fuscoguttatus♀*) (Tan et al. 2019); olive extract (1 g/kg) in common carp (Zemheri-Navruz et al. 2020); and curcumin (120 mg/kg) in common carp (Zhang et al. 2020a). In line with the reports mentioned above, Zhang et al. (2021) demonstrated an increased weight gain and specific growth rate of juvenile common carp fed 60 and 120 mg curcumin per kg for ten weeks and suggested that the observed improvement could be attributed to the enhanced immune response, which increased the growth performance.

Since regulatory functions, such as the metabolism, antioxidant, immune capacity, anti-stress, anti-virus, antibacterial, and anti-parasite activities can directly affect the growth rate, a balanced regulation among these physiological metabolisms seems to be necessary for aquatic species. Hence, these aspects of using medicinal plants in aquaculture must be taken into account.

#### MEDICINAL PLANTS AS ANTI-STRESSORS AND IMMUNOSTIMULANTS

In intensive aquaculture, external cues, such as poor water quality, a high environmental temperature, overcrowding, and pathogen infection, make aquatic animals vulnerable to adverse stress (Asaduzzaman et al. 2009; Chang et al. 2015). The immunomodulatory power of plants is mostly determined by the assessment of the fish immunity via classic biochemical approaches (lysozyme, phagocytic, or respiratory burst activity) or by the study of the immune gene expression (Lysine (Lys), Tumour Necrosis Factor-alpha (TNF-alpha), Interleukin-1 (IL-1), Interleukin-10 (IL-10) genes) (Harikrishnan et al. 2011; Kumar et al. 2013; Chakrabarti et al. 2014). Several studies recommend some natural compounds, including medical plants, to defend against stress induced by external cues (ESM Table S2).

Several herbal plants that originated from terrestrial and marine environments and are considered efficient immunostimulants against viral diseases was confirmed in a study (Chakraborty et al. 2014). In contrast to antibiotics, medical plants as natural resources have outstanding features, such as being highly effective and showing low toxicity and side effects when used correctly (Bone and Mills 2012). The antistress effects of medicinal plants have been mentioned in several studies (Ahmadniaye Motlagh et al. 2019). For instance, an anthraquinone extract from Chinese rhubarb (*Rheum officinale Bail*) improved the tolerance against hyperthermia in freshwater shrimp (*Macrobrachium nipponense*) (Song et al. 2020). Moreover, a sweet wormwood (*Artemisia annua) *leaf extract improved the antioxidant capacity of carp (Taheri Mirghaed et al. 2020) and enhanced the immunity of Nile tilapia (Soares et al. 2020). A methanolic extract of fenugreek (*Trigonella foenum-graecum*) significantly increased the immunity and antioxidative response via improvement of the superoxide dismutase, lysozyme, and phagocytic activities in Nile tilapia (Diab et al. 2023).

The anti-ammonia stress capacity of freshwater prawns was improved by the addition of a moringa (*Moringa oleifera*) leaf extract to the fish’s diet (Kaleo et al. 2019). Furthermore, emodin protected Wuchang bream (*Megalobrama amblycephala*) from crowding stress (Liu et al. 2014), and bupleurum (*Radix Bupleuri*) extracts boosted tilapia against H_2_O_2_-induced oxidative stress (Jia et al. 2019). In common carp, the adverse effects of crowding stress were mitigated using the application of an anthraquinone extract from rhubarb (*Rheum officinale Bail*) (1–2%) (Xie et al. 2008). Also, the anti-stress effect of turmeric in common carp exposed to copper was reported by Rajabiesterabadi et al. (2020).

Several studies have focused on using plant extracts as fish immunostimulants (Prathomya et al. 2019; Rafieepour et al. 2019); according to scientific studies on fish species, the intraperitoneal injection or oral administration of plant extracts enhanced the phagocytic and lysosomal function, respiratory burst and complement activity, as well as the serum protein level (Liu et al. 2010a; Vallejos-Vidal et al. 2016; Balamurugan et al. 2018; Ramezanzadeh et al. 2019). A dietary supplemented by fenugreek (*Trigonella foenum graecum*) seeds (5%) had the highest haemolytic complement and peroxidase activities, indicating that the metabolic and immune status of gilthead seabream (*Sparus aurata* L.) was improved (Guardiola et al. 2018). A supplement of an olive leaf extract (0.1%) in the diet of rainbow trout increased the immune-related gene expression, serum biochemistry parameters, and survival rate (Baba et al. 2018). It is supposed that herbal extracts can positively affect the innate immunity (Devi et al. 2019; de Assis and Urbinati 2020; Soares et al. 2020), adaptive immunity (Abd El-Gawad et al. 2020; Zhang et al. 2020b), mucosal immunity (Van Doan et al. 2019; Ahmadniaye Motlagh et al. 2020a; Ahmadniaye Motlagh et al. 2020b; Heydari et al. 2020; Srichaiyo et al. 2020a; Srichaiyo et al. 2020b), and their anti-stress functions have been proven in several studies (summarised in ESM Table S2).

#### MEDICINAL PLANTS AS AN ANTI-VIRAL AND AN ANTIBACTERIAL TREATMENT

The potential of medicinal plants against a wide range of marine pathogens (bacteria, viruses, fungus, and ectoparasites) was confirmed in *in vitro* and *in vivo* studies (Direkbusarakom et al. 1996; Chitmanat et al. 2005; Ji et al. 2012). The antiviral activity of plant species against aquatic primary pathogenic viruses was reported in different diseases, including white spot syndrome virus (WSSV), grouper iridovirus (GIV), grass carp reovirus (GCRV), spring viraemia of carp virus (SVCV) and cyprinid herpesvirus 2 or 3 (CyHV) (summarised in ESM Table S3). Giant tiger prawn (*Penaeus monodon*) treated with Bermuda grass (*Cynodon dactylon*) displayed no signs of disease and mortality when exposed to white spot syndrome virus (WSSV), while 100% mortality was observed in the control groups (Balasubramanian et al. 2008a; Balasubramanian et al. 2008b). A methanolic extract of fenugreek increased the tolerance of Nile tilapia against *A. hydrophila* (Diab et al. 2023); this could be attributed to the deactivation of the peptidoglycans of *A. hydrophila* due to the increased lysozyme activity that caused enhanced resistance (Masschalck and Michiels 2003; Brott and Clarke 2019). The extract of the plants, such as Bermuda grass (*Cynodon dactylon*), the olive (*olea europaea*), cape jasmine (*Gardenia jasminoides*), and Mexican poppy (*Argemone mexicana*) showed significant anti-virus activity against WSSV through oral administration or injection (Ghosh et al. 2014; Palanikumar et al. 2018; Huang et al. 2019a; Huang et al. 2019b). Furthermore, a berberine hydrochloride (*Clinacanthus nutans*) extract showed a considerable preventive effect against CyHV-2 or CyHV-3 in *Gibel carp* (*Carassius auratus gibelio*) (Su et al. 2021) or koi carp (*Cyprinus carpio koi*) (Haetrakul et al. 2018). Several *in vitro* studies have reported the antibacterial activity in numerous plants against both Gram-positive and Gram-negative marine bacteria (Castro et al. 2008; Roomiani et al. 2013). As summarised in ESM Table S3, some medicinal plants presented specific antibacterial effects on pathogenic bacteria such as the Mongolian milkvetch (*Astragalus membranaceus*) (Wu 2020), fern (*Adiantum capillus-veneris*) (Hoseinifar et al. 2020a), horse mint (*Mentha longifolia*) (Heydari et al. 2020), rosemary (*Rosmarinus officinale*) (Naiel et al. 2020), and prepared foxglove root (*Radix rehmanniae preparate*) (Wu et al. 2019). A tamarind (*Tamarindus indica* L.) pulp extract (15 g/kg) promoted the growth and nutrient digestibility of Nile tilapia, and provided protection against an *A. hydrophila* infection (Adeniyi et al. 2021). The addition of a 1.5 g/kg chinaberry tree (*Melia Azedarach*) extract, a 4–6 g/kg velvet bean (*Mucuna pruriens*) extract, and a 0.5–2 g/kg jojoba extract to the diet improved the resistance against *A. hydrophila* in Catla (*Labeo catla*) (Rajeshwari et al. 2016), Mozambique tilapia (*Oreochromis mossambicus*) (Saiyad Musthafa et al. 2018), and Nile tilapia (Sarhan et al. 2019), respectively. A supplementation of 40% moringa (*Moringa oleifera*) leaf extracts to the diet of carp infected by *A. hydrophila* enhanced the growth, antioxidant and immune response of the carp (Zhang et al. 2020a). Similar promising effects of a medicinal herb supplementation against *A. hydrophila* were observed in different fish species, such as the addition of geniposide in the diet of crucian carp (He et al. 2020), a grape seed extract in the diet of common carp (Mehrinakhi et al. 2021), Mongolian wild onion (*Allium Mongolicum Regel*) (40 mg/kg) in the diet of juvenile snakehead (*Channa argus*) (Li et al. 2018), Smoketree (*Cotinus coggygria*) in the diet of rainbow trout (Bilen and Elbeshti 2019), and marjoram (*Origanum majorana*) (Yousefi et al. 2021) and maidenhair tree (*Ginkgo biloba*) leaf (Bao et al. 2019) extracts in the diet of common carp. The antibacterial activity of several medicinal herbs against *Vibrio* spp. as an opportunistic pathogenic bacteria has been confirmed in many fish species, such as applications of peppermint (*Mentha piperita*) extracts against a *V. harveyi* infection in Barramundi (Talpur 2014), a Chinese rhubarb (*Rheum officinale*) extract (0.1, 1.0 g/kg) against *V. parahaemolyticus* in the orange-spotted grouper (*Epinephelus coioides*) (Kuo et al. 2020), a gale of the wind (*Phyllanthus amarus*) extract (20 g/kg) against *V. alginolyticus* in pacific white shrimp (*Litopenaeus vannamei*) (Ngo et al. 2020), tears of the virgin (*Eleutherine bulbosa*) (12.5 g/kg) against *V. parahaemolyticus* in pacific white shrimp (*Litopenaeus Vannamei*) (Munaeni et al. 2020), and the Chinese herbal medicine, San-Huang-San against *V. parahaemolyticus* in pacific white shrimp (*Litopenaeus vannamei*) (Zhai and Li 2019).

Additionally, the protective roles of some v medicinal herbs against *Streptococcus* spp. as an opportunistic pathogen in aquaculture systems have been mentioned in some scientific studies on Nile tilapia, such as the promising effects of a thumbai (*Leucas aspera*) extract (8 g/kg) against a *Streptococcus agalactiae* infection (Kurian et al. 2020) and an Assam tea (*Camellia sinensis*) extract against *S. agalactiae* (Van Doan et al. 2019). Likewise, the antibacterial activity of some herbs against *Yersinia ruckeri* has been confirmed by incorporating a peppermint (*Mentha Piperita*) extract (Adel et al. 2016), Greek juniper (*Juniperus excelsa*) (Bilen et al. 2021) and coriander (*Coriandrum sativum*) (Naderi Farsani et al. 2019) in the diet of rainbow trout. The functional activity of the plasma lysozyme, blood phagocytes, respiratory burst, and survival rates have improved in Nile tilapia fed a combination of honeysuckle (*Lonicera*) and milkvetch (*Astragalus*) extracts when infected with *A. hydrophila* (Ardo et al. 2008).

In addition, various active ingredients extracted from medicinal plants and essential oils were confirmed to have antibacterial activities against overwhelming bacterial species (Kacaniova et al. 2017). It was found that medicinal plants could show different levels of immune stimulation by injection, immersion, or oral administration (Awad and Awaad 2017).

#### MEDICINAL PLANTS AS AN ANTIPARASITIC

Medicinal plants can be considered as an effective alternative for treating ectoparasites. The antiparasitic activities of medicinal plants, when added to water or administered orally, were confirmed by several studies (Yi et al. 2012; Huang et al. 2013; Fu et al. 2014). A significant improvement in the haematological profile in Nile tilapia challenged by flatworms (*Scutogyrus longicorni*), cichlidogyrus (*Cichlidogyrus thurstone*), cichlidogyrus (*Cichlidogyrus halli*), and monogeneans, cichlidogyrus (*Cichlidogyrus tilapia*) was observed when treated by essential oils extracted from peppermint (*Mentha piperita*) (Hashimoto et al. 2016).

Harikrishnan et al. (2012) reported decreased mortality (40%) and enhanced immunity in the olive flounder (*Paralichthys olivaceus*) infected by the protozoan ciliate *Miamiensis avidus* when a fish-fed diet was supplemented with herbaceous seepweed (*Suaeda maxima*).

Some studies on fish infected with white spot disease (*Ichthyophthirius multifiliis*) showed that ginger (*Zingiber officinale*), sweet wormwood (*Artemisia annua*), tea tree (*Melaleuca alternifolia*), English lavender (*Lavandula angustifolia*), peppermint (*Mentha piperita*), babchi (*Psoralea corylifolia*), orange climber (*Toddalia asiatica*) and Chinese gall (*Galla chinensis*) could potentially control the infection (Zhang et al. 2013; Shan et al. 2014; Song et al. 2015; Valladao et al. 2016; Wu et al. 2017; de Freitas Souza et al. 2019; Fu et al. 2019). The white spot disease (*Ichthyophtirius multifiliis*) in the sailfin molly (*Poecilia latipinna*) was also inhibited by a mother worth (*Matricaria chamomilla*) extract (Gholipourkanani et al. 2012). 100% *in vivo* efficacy against monogenean *Dactylogyrus intermedium* was reported in infected goldfish (*Carassius auratus*) treated by a methanol extract of bupleurum root (*Radix bupleuri chinensis*), aqueous and methanol extracts of cinnamon (*Cinnamomum cassia*), a methanol extract of Chinese spice bush (*Lindera aggregata*) and methanol and ethyl acetate extracts of golden larch (*Pseudolarix kaempferi*) (Wu et al. 2011; Ji et al. 2012). The oral administration of an aqueous extract of rosemary (*Rosmarinus officinalis*) could kill gill fluke (*Dactylogyrus minutus*) in common carp (Zoral et al. 2017). A bath treatment of guppies [*Poecilia reticulata* (Peters)] using an ethanolic extract of *ginger* (*Zingiber Officinale*) significantly decreased the salmon fluke (*Gyrodactylus turnbulli*) infection (Levy et al. 2015). More and more herbal medicines can be found to be used in parasite remedies of fish species (see ESM Table S4).

## CONCLUSION

Medicinal plants exhibit a promising potential for the current needs of the intensive and largescale production in the aquaculture industry and are a substitute for chemotherapy in the treatment of disease outbreaks and control of farmed fish diseases. Studies have shown they are a proper candidate for disease prevention and control guidelines to develop pollution-free aquaculture and green aquatic products. Each medicinal plant is specific in its action against infectious diseases, and the dose of a specific medicinal plant is a crucial factor in the efficacy against specific infectious agents. However, the toxicological safety research of medicinal plants needs to be further studied and more research is also required to elucidate upon plant products and their modes of action and to test the plant effects on an organism’s physiology in order to establish appropriate treatment strategies. In this context, researchers may benefit from the traditional knowledge of fish farmers who regularly use plants. Moreover, research is still relatively scarce on the use of some compatible herbs with a synergistic effect. Therefore, the effects of some compatible herbs on the health status of fish and shellfish might be an exciting topic in aquaculture.
