# Supplementary material for: An update about beneficial effects of medicinal plants in aquaculture: A review
Source: Vet Med (Praha). 2023 Dec 26;68(12):449–63. doi: 10.17221/96/2023-VETMED (PMC10828785; doi:10.17221/96/2023-VETMED)
Supplement: Supplementary Tables [file VETMED-68-12-123096-s001.pdf]

https://doi.org/10.17221/96/2023-VETMED

An update about beneficial effects of medicinal plants in aquaculture: A review

Faranak Dadras, Josef Velisek, Eliska Zuskova*

Faculty of Fisheries and Protection of Waters, South Bohemian Research Center of Aquaculture and Biodiversity of Hydrocenoses, Research Institute of Fish Culture and Hydrobiology, University of South Bohemia in Ceske Budejovice, Ceske Budejovice, Czech Republic

**Corresponding author: fdadrasasiabar@frov.jcu.cz*

The authors are fully responsible for both the content and the formal aspects of the electronic supplementary material. No editorial adjustments were made.

Electronic Supplementary Material (ESM)

Table S1. Use of medicinal plants as growth promoters in aquaculture

Table S2. Use of medicinal plants as anti-stress and immunostimulants in aquaculture

Table S3. Use of medicinal plants as anti-virus and anti-bacteria in aquaculture

Table S4. Use of medicinal plants as anti-parasite in aquaculture

List of references

Veterinarni Medicina, 68, 2023 (12): 449–463

https://doi.org/10.17221/96/2023-VETMED

Table S1. Use of medicinal plants as growth promoters in aquaculture

Herbal name	Compound	Dose	Fish species	Function	Reference
Garlic and ginger	Garlic : ginger = 1 : 1	2%	Beluga (*Huso huso*)	Improve growth and globulin	Gholipour Kanani et al. (2014)
Gokhru (*Pedalium murex*)	Extract	0.1%	Rohu (*Labeo rohita*)	Improve weight gain and specific growth ratio	Ojha et al. (2014)
Alder buckthorn (*Frangula*) bark Emodin	Emodin	30 mg/kg	Rohu (*Labeo rohita*)	Improve growth performance and immunity	Giri et al. (2016)
Ginger (*Zingiber officinale*)	Powder	0.8%	Rohu (*Labeo rohita*)	Improve growth performance	Sukumaran et al. (2016)
Common purslane (*Portulaca oleracea* L.)	Leaf	powder 1–3%	Nile tilapia (*Oreochromis niloticus*)	Improve growth, antioxidant, and immunological responses	Abdel-Razek et al. (2019)
Mojave yucca (*Yucca schidigera*)	Liquid extract	0.75 mg/l	European seabass (*Dicentrarchus labrax*)	Benefit the growth, improve the physiological responses of and decrease the mortality rates	Fayed et al. (2019)
Maidenhair tree (*Ginkgo biloba*)	Extract	0.1–0.2%	Rainbow trout (*Oncorhynchus mykiss*)	Moderate the growth-suppressing effects, stress, and liver tissue damage induced by diazinon	Hajirezaee et al. (2019)
Ferula (*Ferula asafoetida*)	Powder	20–25 g/kg	Common carp (*Cyprinus carpio*)	Improve growth performance	Safari et al. (2019)
Turmeric (*Curcuma longa*)	Powder	4%	Nile tilapia (*Oreochromis niloticus*)	Improve growth performance	Sanchez et al. (2019)
Chaff flower (*Achyranthes aspera*)	Powder	0.5%	Rohu (*Labeo rohita*)	Improve growth performance	Sharma et al. (2019)
Onion (*Allium cepa*)	Powder	10 g/kg	White carp (*Cirrhinus mrigala*)	Improve growth performance	Sikotariya (2019)
Dandelion (*Taraxacum officinale*)	Extract	0.8%	Common carp (*Cyprinus carpio*)	Improve growth performance	Sirakov et al. (2019)
Fluted pumpkin (*Telfairia occidentalis*)	Extract	1%	African sharp-tooth cat-fish (*Clarias gariepinus*)	Improve growth performance	Ta et al. (2019)
Wolfberry (*Lycium barbarum*)	Extract	0.5–2%	Hybrid grouper	Improve growth performance	Tan et al. (2019)

Veterinarni Medicina, 68, 2023 (12): 449–463

https://doi.org/10.17221/96/2023-VETMED

Table S1 to be continued

Herbal name	Compound	Dose	Fish species	Function	Reference
Dandelion	Extract	1 g/kg	Silverfish (*Trachinotus ovatus*)	Improve growth performance	Tan and Sun (2020)
Aloe barbadensis miller (*Aloe vera*)	Extract	0.4%	Common carp (*Cyprinus carpio*)	Improved growth performance, blood parameters, antioxidant capacity	Jafarinejad et al. (2020); Lamichhane et al. (2020)
Climbing Senecio (*Senecio scandens* buch-ham)	Extract	0.05–0.1%	Hybrid grouper	Improve growth performance	Sun et al. (2020)
Salidroside	Powder	100 mg/kg	Common carp (*Cyprinus carpio*)	Improve growth performance	Yang et al. (2020)
Hardy Rubber Tree (*Eucommia ulmoides*)	Extract	40 g/kg	Rainbow trout (*Oncorhynchus mykiss*)	Improve growth performance	Yao et al. (2020)
Olive extract	Extract	1 g/kg	Common carp (*Cyprinus carpio*)	Improve growth performance	Zemheri-Navruz et al. (2020)
Curcumin	Curcumin	120 mg/kg	Common carp (*Cyprinus carpio*)	Improve growth performance	Zhang et al. (2021)
Jamun (*Syzygium cumini*)	Leaf extract	10 g/kg	Nile tilapia (*Oreochromis niloticus*)	Improve growth performance	Kannan et al. (2022)
Sweet wormwood (*Artemisia annua*)	Extract	0.05%	Largemouth bass (*Micropterus salmoides*)	Improve growth performance	He et al. (2022)
Fnugreek (*Trigonella foenum-graecum*)	Methanolic Extract	0.1%	Nile tilapia (*Oreochromis niloticus*)	Improve growth performance	Diab et al. (2023)

Veterinarni Medicina, 68, 2023 (12): 449–463

https://doi.org/10.17221/96/2023-VETMED

Table S2. Use of medicinal plants as anti-stress and immunostimulants in aquaculture

Herbal name	Compound	Dose	Species	Function	References
Anthraquinone	Extract	1–2%	Common carp (*Cyprinus carpio*)	Mitigate negative effects of crowding stress	Xie et al. (2008)
Anthraquinone	Extract	0.1–0.2%	Giant river prawn (*Macrobrachium rosenbergii*)	Prevent high-temperature stress	Liu et al. (2010)
Garlic (*Allium sativum*) and Ginger (*Zingiber officinale*)	Garlic : ginger = 1 : 1	2%	Beluga (*Huso huso*)	Improve growth, globulin	Gholipour Kanani et al. (2014)
Curcumin	Curcumin	60 mg/kg	Wuchang bream (*Megalobrama amblycephala*)	Improve non-specific immunity (IL-1β, TNF-α)	Xia et al. (2015)
Peppermint (*Mentha piperita*)	Menthone, menthol	3%	Caspian white fish (*Rutilus frisii kutum*)	Improve immunity (both mucosal and systemic)	Adel et al. (2015)
Blue Spade Flower (*Hybanthus enneaspermus*)	Extract	3 g/kg	Rohu (*Labeo rohita*)	Enhance haematological, biochemical, and immune responses and the intestinal enzymatic activities	Giri et al. (2017)
Basil (*Ocimum gratissimum*)	Leaf extract	12 g/kg	African sharp-tooth catfish (*Clarias gariepinus*)	Elevate antioxidant and immuno-activity (activities of AST, ALT, ALP, urea, creatinine levels)	Abdel-Tawwab et al. (2018)
Garlic (*Allium sativum*)	Extract	1–2%	Rainbow trout (*Oncorhynchus mykiss*)	Induce changes in the intestinal microbiota, conferring beneficial effects on the host	Buyukdeveci et al. (2018)
Mongolian wild onion (*Allium mongolicum Regel*)	Flavonoids	40 mg/kg	Northern snakehead (*Channa argus*)	Improve innate immunity (SOD, IgM, HSP70, HSP90, IκB-α, GR)	Li et al. (2018)
Clove (*Eugenia caryophyllata*), buds	Extract	15 g/kg	African sharptooth catfish (*Clarias gariepinus*)	Improve immunity (increase red blood cells, haemoglobin, haematocrit, white blood cells, platelets, lymphocytes, and heterocysts, while monocytes, eosinophil)	Adeshina et al. (2019)
Fishwort (*Houttuynia cordata*)	Powder	1.5–3%	Sea cucumber (*Apostichopus japonicus*)	Improve immunity (increase alkaline phosphatase, acid phosphatase, superoxide dismutase, lysozyme activity of intestine)	Dang et al. (2019)
Rosemary (*Salvia Rosmarinus*)	Leaf powder	2–3%	Common carp (*Cyprinus carpio*)	Mitigate the negative effects of crowding stress	Yousefi et al. (2019)

https://doi.org/10.17221/96/2023-VETMED

Table S2 to be continued

Herbal name	Compound	Dose	Species	Function	References
Female ginseng (*Angelica sinensis*)	Extract	1–6 g/kg	Common carp (*Cyprinus carpio* var. Jian)	It could be used as an inhibitor of trichlorfon stress in fish	Li et al. (2019a)
Female ginseng (*Angelica sinensis*)	Extract	1–6 g/kg	Common carp (*Cyprinus carpio* var. Jian)	Could be used as a natural antioxidant	Li et al. (2019b)
Bupleurum (*Radix Bupleuri*)	Saikosaponins and flavonoids	1–3 g/kg	Nile tilapia (*Oreochromis niloticus*)	Protect against H_2O_2-induced oxidative damage	Jia et al. (2019)
Moringa (*Moringa oleifera*)	Seeds and leaves extract	7, 10%	Nile tilapia (*Oreochromis niloticus*)	Resist oxidative stress	Ibrahim et al. (2019)
Moringa (*Moringa oleifera*)	Leaf powder	0.5%	Giant river prawn (*Macro-brachium rosenbergii*)	Prevent high ammonia stress	Kaleo et al. (2019)
Common purslane (*Portulaca oleracea* L.)	Leaf powder	1–3%	Nile tilapia (*Oreochromis niloticus*)	Improve growth, antioxidant, and immunological responses	Abdel-Razek et al. (2019)
Maidenhair tree (*Ginkgo biloba*)	Extract	0.1–0.2%	Rainbow trout (*Oncorhynchus mykiss*)	Moderate the growth-suppressing effects, stress, and liver tissue damage induced by diazinon	Hajirezaee et al. (2019)
Aloe-emodin	Aloe-emodin	10 mg/kg	Rohu (*Labeo rohita*)	Improve innate immunity (IL-1β, IL-8, TNF-α, iNOS)	Devi et al. (2019)
Hardy rubber tree (*Eucommia ulmoides*)	Leaf powder	5–20 g/kg	Turbot (*Scophthalmus maximus* L.)	Improve non-specific immunity (lysozyme, immune cytokines)	Zhang et al. (2019)
Icariin	Icariin	100 mg/kg	Chinese mitten crab (*Eriocheir sinensis*)	Improve non-specific immunity (transcrip-tional expression of MnSOD, Trx-1, Relish)	Zheng et al. (2019)
Milkvetch (*Astragalus*) polysaccharide	Astragalus polysaccharide	800 mg/kg	Sea cucumber (*Apostichopus japonicus*)	Improve non-specific immunity (NF-κB signaling pathway)	Song et al. (2019)
Mangrove apple (*Sonneratia*)	Leaf extract	3.0–5.0%	Goldfish (*Carassius auratus*)	Improve non-specific immunity (albumin, lysozyme activity)	Afzali and Wong (2019)
Elephant's Foot (*Elephantopus scaber*)	Extracts	5 g/kg	Nile tilapia (*Oreochromis niloticus*)	Improve mucosal immunity (skin mucus immune response, serum immunity)	Van Doan et al. (2019b)
Aloe barbadensis (*Aloe vera*)	Powder	15 g/kg	Rainbow trout (*Oncorhynchus mykiss*)	Improve immunity (respiratory burst activity, lysozyme activity, and comple-ment system; TNF-α, IL-1β, IL-6, IL-8 gene expression)	Mehrabi et al. (2019)

https://doi.org/10.17221/96/2023-VETMED

Table S2 to be continued

Herbal name	Compound	Dose	Species	Function	References
Fingerroot (*Boesenbergia rotunda*)	Powder	10 g/kg	Nile tilapia (*Oreochromis niloticus*)	Improve immunity (lysozyme and peroxidase activities)	Van Doan et al. (2019a)
Rosemary (*Rosmarinus Officinalis*)	Rosemary	0.5%	Nile tilapia (*Oreochromis niloticus*)	Improve immunity (complement C3, IgM)	Naiel et al. (2019)
Lemon peels	Powder	1–2%	Nile tilapia (*Oreochromis niloticus*)	Improve immunity (lysozyme and MPO activities, nitric oxide content, phagocytic activity)	Rahman et al. (2019)
Milk thistle (*Silybum marianum*)	Seed powder	7.5 –10 g/kg	Nile tilapia (*Oreochromis niloticus*)	Improve immunity (IgM, SOD, CAT)	Hassaan et al. (2019)
Assam tea (*Camellia sinensis*)	Extract	2 g/kg	Nile tilapia (*Oreochromis niloticus*)	Improve immunity (serum lysozyme, peroxidase, ACH50, phagocytosis, respiratory burst activities)	Van Doan et al. (2019c)
Garlic (*Allium sativum*)	Powder	15–20 g/kg	Nile tilapia (*Oreochromis niloticus*)	Improve immunity (haemoglobin, haematocrit, white blood cells, red blood cells, mean corpuscular volume, mean corpuscular haemoglobin, mean corpuscular haemoglobin concentration)	Naqi et al. (2019)
Fenugreek (*Trigonella foenum-graecum*)	Seed extract	3–5%	Nile tilapia (*Oreochromis niloticus*)	Improve immunity (IL-6, IL-8 genes expressions)	Abbas et al. (2019)
Common barberry (*Berberis vulgaris*)	Root powder and methanol extract	500 mg/kg	Rainbow trout (*Oncorhynchus mykiss*)	Improve immunity (lysozyme, ACH50)	Ramezanzadeh et al. (2019)
Maidenhair tree (*Ginkgo biloba*)	Leaf ethanol extract	1–2 g/kg	Rainbow trout (*Oncorhynchus mykiss*)	Improve immunity (peroxidase activity, total immunoglobulin, and lysozyme activity; IL-1β, TGF-β1 expression)	Hajirezaee et al. (2019)
Moringa (*Moringa oleifera*), Rosemary (*Rosmarinus officinalis*), Turmeric (*Curcuma longa*)	Leaf powder	1%	Nile tilapia (*Oreochromis niloticus*)	Improve immunity (lysozyme and respiratory burst activity)	Ayoub et al. (2019)

https://doi.org/10.17221/96/2023-VETMED

Table S2 to be continued

Herbal name	Compound	Dose	Species	Function	References
Maidenhair tree (*Ginkgo biloba*)	Leaf extract	10 g/kg	Common carp (*Cyprinus carpio*)	Improve immunity by upregulate IL-1β, IL-8, TNF-α, IL-10, TGF-β	Bao et al. (2019)
Guava (*Psidium guajava*)	Leaf	0.5–1%	Common carp (*Cyprinus carpio*)	Improve immunity (total Ig, alkaline phosphatase activity, lysozyme activity, TNF-α, IL-1β, IL-8)	Hoseinifar et al. (2019)
Oregano (*Origanum vulgare*)	Powder	0.5–1%	Gilt-head seabream (*Sparus aurata*)	Improve both humoral (IgM, bactericidal activity in skin mucus and protease activity in serum), cellular (head kidney leucocytes phagocytic ability) immunity	Beltran et al. (2020)
Prepared foxglove root (*Radix Rehmanniae Preparata*)	–	0.2%	Bulatmai barbel (*Luciobarbus capito*)	Improve immunity (increase serum lysozyme, ACP, SOD, ALP, TP)	Wu et al. (2019)
Mexican tea (*Chenopodium ambrosioides* L.)	Powder	0.5–2%	Pacific red snapper (*Lutjanus peru*)	Improve non-specific immunity (lysozyme and peroxidase activities)	Maldonado-Garcia et al. (2019)
Ginger (*Zingiber officinale*)	Powder	2%–3%	Zebrafish (*Danio rerio*)	Elevate antioxidant and immuno-activity (decrease mmunoglobulin level, alternative complement activity, lysozyme activity, up-regulate catalase, lysozyme mRNA levels)	Ahmadifar et al. (2019)
Mushroom	Extract	6%	Spiral Babylon (*Babylonia spirata*)	Enhance antioxidants (SOD, CAT)	Chelladurai and Maran (2019)
Mojave yucca (*Yucca schidigera*)	Liquid extract	0.75 mg/l	European seabass (*Dicentrarchus labrax*)	Improve the growth, improve the physiological responses, and decrease the mortality rates	Fayed et al. (2019)
Lemon	Dehydrated peel	1.5–3%	Gilt-head seabream (*Sparus aurata*)	Improve the biomolecular markers related to general stress, oxidative stress, apoptosis	Garcia Beltran et al. (2019)
Mix Fingerroot (*Boesenbergia Pandurata*), Indian nightshade (*Solanum ferox*) and Pinecone ginger (*Zingiber zerumbet*)	Plant extracts 1 : 1 : 1	3–5% of fish body weight	Nile tilapia (*Oreochromis niloticus*)	Enhance the immunomodulatory activity (white blood cell, red blood cell, phagocytic index)	Hardi et al. (2019)
Gotu kola (*Centella asiatica*)	Leaf and stem extract	10 g/kg	Nile tilapia (*Oreochromis niloticus*)	Improve mucosal immunity (serum lysozyme, serum peroxidase activities)	Srichaiyo et al. (2020b)

Veterinarni Medicina, 68, 2023 (12): 449–463

https://doi.org/10.17221/96/2023-VETMED

Table S2 to be continued

Herbal name	Compound	Dose	Species	Function	References
Horse mint (*Mentha longifolia*)	Stem, leaf and flower	0.2%	Rainbow trout (*Oncorhynchus mykiss*)	Improve mucosal immunity (lysozyme and TNF-α expression)	Heydari et al. (2020)
Fishwort (*Houttuynia cordata*)	Leaf and stem powder	10 g/kg	Nile tilapia (*Oreochromis niloticus*)	Increase mucosal immunity (serum lysozyme, peroxidase, ACH50, phagocytosis)	Srichaiyo et al. (2020a)
Garlic (*Allium sativum*)	Extract	0.15 ml/kg	Guppy (*Poecilia reticulata*)	Improve mucosal immunity (lysozyme, ACH50, total IgG, ALP activity)	Ahmadniaye Motlagh et al. (2020)
Olive (*Olea europea* L.)	Olive waste cake	2.5 g/kg	Rainbow trout (*Oncorhynchus mykiss*)	Improve mucosal immunity (IL-1β, IL-8, TNF-α, TGF-1β expression)	Hoseinifar et al. (2020c)
Moringa (*Moringa oleifera*)	Leaf extract	1.5, 5%	Nile tilapia (*Oreochromis niloticus*)	Improve immunity (respiratory burst, phagocytic and lysozyme activities, IgM level)	Abd El-Gawad et al. (2020)
Rosemary (*Salvia rosmarinus*)	Leaf powder	10 g/kg	Nile tilapia (*Oreochromis niloticus*)	Improve immunity (serum catalase, alternative complement, nitroblue tetrazolium, lysozyme activities)	Naiel et al. (2020)
Fenugreek (*Trigonella foenum-graecum*)	Seed powder	3%	Nile tilapia (*Oreochromis niloticus*)	Improve immunity (IL-1β, TNF-α expression, AST, ALT activity)	Moustafa et al. (2020)
Thumbai (*Leucas aspera*)	Powder	8 g/kg	Nile tilapia (*Oreochromis niloticus*)	Improve immunity (lysozyme activities in the serum, mucus, serum peroxidase, phagocytosis activity)	Kurian et al. (2020)
Nettle (*Urtica dioica*)	Leaf powder	0.5%	Rainbow trout (*Oncorhynchus mykiss*)	Improve immunity (TNF-α, IL-1β, L-6, IL-8 expression)	Mehrabi et al. (2020)
Chinese yam (*Dioscorea oppositifolia*)	Water extract	0.4%	Rainbow trout (*Oncorhynchus mykiss*)	Improve immunity (serum C4 level, lysozyme activity, IL-6, TNF-α, IL-2 content; intestinal GPx1, TNF-α, factor H, HSP90BA, HSC70A expression)	Wang et al. (2020)
Indian jujube (*Ziziphus mauritiana*)	Leaf powder	10 g/kg	Rainbow trout (*Oncorhynchus mykiss*)	Improve immunity (lysozyme, IL-1β expression)	El Asely et al. (2020)
Common water hyacinth (*Eichhornia crassipes*)	Leaf extract	1%	Rainbow trout (*Oncorhynchus mykiss*)	Improve immunity (Ig, ACH50, respiratory burst activity)	Rufchaei et al. (2020)

Veterinarni Medicina, 68, 2023 (12): 449–463
https://doi.org/10.17221/96/2023-VETMED

Table S2 to be continued

Herbal name	Compound	Dose	Species	Function	References
Ribwort Plantain (*Plantago lanceolata*)	Leaf powder	1–3 g/kg	Rainbow trout (*Oncorhynchus mykiss*)	Improve immunity (oxidative radical production, lysozyme activity, total myeloperoxidase)	Elbesthi et al. (2020)
Lemon verbena (*Aloysia citrodora*)	Leaf powder	2%	Rainbow trout (*Oncorhynchus mykiss*)	Improve immunity (lysozyme activity, total immunoglobulin level, IL-1β, IL-8, TNF-α, TGF-β expression)	Hoseinifar et al. (2020b)
Lemon balm (*Melissa officinalis*)	Methanol extract	0.5–1 g/kg	Rainbow trout (*Oncorhynchus mykiss*)	Improve immunity (total myeloperoxidase)	Bilen et al. (2020)
Rosemary (*Rosmarinus officinalis*)	Leaf ethanol extract	1–3 g/kg	Rainbow trout (*Oncorhynchus mykiss*)	Improve immunity (lysozyme, total immunoglobulin)	Karatas et al. (2020)
Aloe barbadensis miller (*Aloe vera*)	Extracts	0.5, 1.0, 2.0%	Small-scaled pacu (*Piaractus mesopotamicus*)	Improve innate immunity (hormones like cortisol)	de Assis and Urbinati (2020)
Sweet wormwood (*Artemisia annua*)	Alcohol extract	0.5%	Nile tilapia (*Oreochromis niloticus*)	Improve innate immunity (respiratory burst of leukocyte, lysozyme activity)	Soares et al. (2020)
Moringa (*Moringa oleifera*)	Leaf powder	150 g/kg	Grass carp (*Ctenopharyngodon idella*)	Improve immunity (TNF-α, IL-8, and IFN-γ expression)	Faheem et al. (2020)
Common purslane (*Portulaca oleracea* L.)	Leaf ethanol extract	0.5%	Grass carp (*Ctenopharyngodon idella*)	Improve immunity (total immunoglobulin level, lysozyme activity)	Ahmadifar et al. (2020)
Common mallow (*Malvae sylvestris*)	Flower aqueous extract	3–5%	Rainbow trout (*Oncorhynchus mykiss*)	Improve immunity (ACH50, total immunoglobulin, lysozyme)	Rashidian et al. (2020)
Oak (*Quercus castaneifolia*)	Leaf extract	1–2 g/kg	Common carp (*Cyprinus carpio*)	Improve immunity (reduced glutathione, lysozyme, complement, bactericidal activity, decreased malondialdehyde)	Paray et al. (2020)
Garlic (*Allium sativum*)	Extract	5 g/kg	Common carp (*Cyprinus carpio*)	Improve immunity (lysozyme, IgM, RBC, haemoglobin, haematocrit)	Karimi Pashaki et al. (2020)
Sage (*Salvia officinalis*) and Lemon verbena (*Lippia citriodora*) extracts	Extract	0.1%	Gilt-head seabream (*Sparus aurata*)	Improve immunity (upregulate lysozyme, IgM, TNF-α, IL-1β, TNF-β1, IL-10)	Salomon et al. (2020)
Ttears of the virgin (*Eleutherine bulbosa*)	Powder	12.5 g/kg	Pacific white shrimp (*Litopenaeus vannamei*)	Improve immunity (total haemocyte count, phenoloxidase activity, and respiratory bursts)	Munaeni et al. (2020)

https://doi.org/10.17221/96/2023-VETMED

Table S2 to be continued

Herbal name	Compound	Dose	Species	Function	References
Geniposide	-	100 mg/kg	Crucian carp (*Carassius carassius*)	Improve immunity (increase lysozyme activity, SOD activity, C3, CAT activity)	He et al. (2020)
Horse mint (*Mentha longifolia*)	Extract	4–6%	Caspian white fish (*Rutilus frisii kutum*)	Improve immunity (increase lysozyme)	Gholamhosseini et al. (2020a)
Mongolian milkvetch (*Astragalus membranaceus*) nd Bupleurum (*Bupleurum chinense*)	-	0.25–0.5%	Pacific white shrimp (*Litopenaeus vannamei*)	Improve immunity (expression of IMD, lysozyme, Toll-like receptor)	Angela et al. (2020)
Black cumin (*Nigella sativa*)	Extract	7.5 g/kg	Pacific white shrimp (*Litopenaeus vannamei*)	Improve immunity (Total Haemocyte Count, granular cells, hyalin cells, relative percent survival)	Nur et al. (2020)
Doum Palm (*Hyphaene thebaica*)	Fruit powder	10–15 g/kg	African sharp tooth catfish (*Clarias gariepinus*)	Improve non-specific immunity (lysozyme, nitric oxide production, and sialoglycans, namely α 2,3-sialyltransferase. α 2,6-sialyltransferase content)	Al-Khalaifah et al. (2020)
Peppermint (*Mentha piperita*)	Powder	4 g/kg	Caspian roach (*Rutilus caspicus*)	Improve immunity (increase soluble protein, alkaline hosphatase, lysozyme enzyme activity)	Paknejad et al. (2020)
Horse mint (*Mentha longifolia*)	Leaf	40%	Gibel carp (*Carassius auratus gibelio*)	Improve immunity (increase SOD, CAT, and lysozyme activities, or decreased serum MDA, and PCC contents)	Zhang et al. (2020a)
Gale of the wind (*Phyllanthus amarus*)	Extract	20 g/kg	Pacific white shrimp (*Litopenaeus vannamei*)	Improve immunity (total haemocyte count, PO activity, phagocytic activity, O_2^- production)	Ngo et al. (2020)
Garlic (*Allium sativum*)	Extract	2–4%	Pacific white shrimp (*Litopenaeus vannamei*)	Enhance the activities of antioxidant	Chirawithayaboon et al. (2020)
Milkvetch (*Astragalus*) polysaccharides	–	0.1–0.2%	Chinese mitten crab (*Eriocheir sinensis*)	Improve immunity (phagocytic activity of haemocytes and the expression levels of the PO, GSH-Px, ALF, Crus1, MasL, HSP genes)	Cui et al. (2020)
Pomegranate (*Punica granatum*)	Peel extract	2%	Goldfish (*Carassius auratus*)	Reduce the enteric gram-negative bacteria count	Ahmadifar et al. (2020)

Veterinarni Medicina, 68, 2023 (12): 449–463

https://doi.org/10.17221/96/2023-VETMED

Table S2 to be continued

Herbal name	Compound	Dose	Species	Function	References
Rock-tea (*Jasonia glutinosa*)	Powder	10–30%	Gilt-head seabream (*Sparus aurata*)	Have antioxidant protection and immunostimulant effects (peroxidase activity, immunoglobulin M levels, complement activity, phagocytic, respiratory burst, peroxidase activities)	Espinosa et al. (2020)
Anthraquinone	Water extracts	0.25–0.5 g/kg	Freshwater shrimp (*Macrobrachium nipponense*)	Improve hyperthermia tolerance	Song et al. (2020)
Turmeric (*Curcuma longa*)	–	10 g/kg	Common carp (*Cyprinus carpio*)	Improve anti-stress against copper exposure	Rajabiesterabadi et al. (2020)
Sweet wormwood (*Artemisia annua*)	Leaf extract	0.5%	Nile tilapia (*Oreochromis niloticus*)	Improve innate immunity (respiratory burst of leukocyte, lysozyme activity)	Soares et al. (2020)
Mojave yucca (*Yucca schidigera*)	Extract	0.75 mg/l	Common carp (*Cyprinus carpio*)	Anti-inflammation influences induced by acute ammonia stress, improve growth and antioxidant (CAT, SOD, IL-10), decrease TNF-α, IFN-γ, HSP-70, IL-1β	Dawood et al. (2021)
Allicin	Allicin	1 g/kg	Nile tilapia (*Oreochromis niloticus*)	Improve innate immunity (IgG and IgM)	Hamed et al. (2021)
Siberian ginseng (*Acanthopanax senticosus*)	Powder	2–4 %	Nile tilapia (*Oreochromis niloticus*)	Improve immunity (plasma and hepatic non-specific immune indexes)	Li et al. (2021)
Licorice (*Glycyrrhiza glabra* L.)	Root powder	10 g/kg	Nile tilapia (*Oreochromis niloticus*)	Improve immunity (lysozyme activity, respiratory burst, total immunoglobulin)	Abdel-Tawwab and El-Araby (2021)
Wild tarragon (*Artemisia dracunculus*)	Methanol extract	2%	Rainbow trout (*Oncorhynchus mykiss*)	Improve immunity (ACH50, mucus bactericidal activity)	Gholamhosseini et al. (2021)
Milkvetch (*Astragalus*) polysaccharides	Powder	1 g/kg	Grass carp (*Ctenopharyngodon idella*)	Improve immunity (TNF-α, IL-1β, CSF, ILF2, DCLK1, TAGAP expression)	Shi et al. (2021)
Marjoram (*Origanum majorana*)	Extract	200 mg/kg	Common carp (*Cyprinus carpio*)	Improve immunity (increase lysozyme, total immunoglobulin levels, mucosal complement, lysozyme. alkaline phosphatase activities)	Yousefi et al. (2021)
Grape seed (*Vitis vinifera*)	Extract	20–30 g/kg	Common carp (*Cyprinus carpio*)	Improve immunity (increase serum total protein, globulin, lysozyme activity)	Mehrinakhi et al. (2021)

Veterinarni Medicina, 68, 2023 (12): 449–463

https://doi.org/10.17221/96/2023-VETMED

Table S2 to be continued

Herbal name	Compound	Dose	Species	Function	References
Jamun (*Syzygium cumini*)	Leaf extract	10 g/kg	Nile tilapia (*Oreochromis niloticus*)	Increase immunological antioxidant parameters	Kannan et al. (2022)
Fenugreek (*Trigonella foenum-graecum*)	Methanolic extract	0.2%	Nile tilapia (*Oreochromis niloticus*)	Increase antioxidant enzyme, lysozyme, phagocytic activities	Diab et al. (2023)

IL-1β = Interleukin-1beta; TNF-α = Tumor Necrosis Factor-alpha; AST = Aspartate transaminase; ALT = Alanine transaminase; ALP = Alkaline phosphatase; SOD = Superoxide dismutase; IgM = Immunoglobulin M; HSP70 = Heat Shock Protein 70; HSP90 = Heat Shock Protein 90; IκB-α = nuclear factor of kappa light polypeptide gene enhancer in B-cells inhibitor, alpha; GR = Glucocorticoid Receptor; H_2O_2 = Hydrogen peroxide; IL-8 = Interleukin-8; iNOS = Inducible Nitric Oxide Synthase; MnSOD = Manganese superoxide dismutase; Trx-1 = Thioredoxin 1; NF-κB = Nuclear factor kappa B; IL-6 = Interleukin-6; C3 = Complement Component 3; MPO = Myeloperoxidase; CAT = Catalase; ACH50 = Alternative Complement Activity; TGF-β1 = Transforming Growth Factor beta1; IL-10 = Interleukin-10; ACP = Acid Phosphatase; TP = total protein; mRNA = Messenger Ribonucleic acid; IgG = Immunoglobulin G; C4 = Complement Component 4; IL-2 = Interleukin-2; GPx = Glutathione Peroxidase; HSC70 = Heat Shock cognate 70; Ig = Immunoglobulin; IFN-γ = Interferon Gamma; RBC = Red Blood Cell; IMD = Immune Deficiency; MDA = Malondialdehyde; PCC = Prothrombin Complex Concentrate; O_2^- = Superoxide anion radical; PO = Phenoloxidase; GSH-Px = Glutathione peroxidase; ALF = TFIIAalpha/beta-like factor; Crus1 = Crustin 1; MasL = Maackia Amurensis Seed Lectin; HSP = Hereditary Spastic Paraplegia; CSF = Cerebrospinal Fluid; ILF2 = Interleukin enhancer-binding factor 2; DCLK1 = Doublecortin Like Kinase 1; TAGAP = T cell activation RhoGTPase activating protein

Veterinarni Medicina, 68, 2023 (12): 449–463

https://doi.org/10.17221/96/2023-VETMED

Table S3. Use of medicinal plants as anti-virus and anti-bacteria in aquaculture

Anti-virus					
Herbal name	Compound	Dose	Fish species	Virus	References
Mexican poppy (*Argemone mexicana*)	Ethyl acetate extracts of stem and root	100–400 mg/kg	Pacific white shrimp (*Penaeus vannamei*)	WSSV	Palanikumar et al. (2018)
Sabah Snake Grass (*Clinacanthus nutans*)	Extract	0.1–2.5 mg/ml	Common carp (*Cyprinus carpio* koi)	CyHV-3	Haetrakul et al. (2018)
Magnolol	–	0.62–2.46 mg/l	Grass carp (*Ctenopharyngodon idella*)	GCRV	Chen et al. (2018)
Coumarin derivative	–	10 µl/fish	Zebrafish (*Danio rerio*)	SVCV	Shen et al. (2018)
Saikosaponin D	–	6 mg/kg	Common carp (*Cyprinus carpio*)	SVCV	Shen et al. (2019)
Cape jasmine (*Gardenia jasminoides*)	Extract	100 µl/fish	Red swamp crayfish (*Procambarus clarkii*)	WSSV	Huang et al. (2019a, 2019b)
Hesperetin	–	40–60 mg/kg	Red swamp crayfish (*Procambarus clarkii*)	WSSV	Qian and Zhu (2019)
Plant mixture	Mixture of *Allium sativum* (40%), *Zingiber officinale* (20%), Eastern purple coneflower (*Echinacea purpurea*) (20%), and Holy basil (*Ocimum sanctum*) (20%)	4 g/kg	Pacific white shrimp (*Penaeus vannamei*)	WSSV	Fierro-Coronado et al. (2019)
Chinese Violet (*Viola philippica*)	Aqueous extract	1 mg/fish	*Epinephelus* spp.	GIV	Yu et al. (2019)
Star anise (*Illicium verum* Hook. f.)	Extract	125–500 µg/ml	Grouper	GIV	Liu et al. (2020)
Olive (*Olea europaea*)	Methanolic extract	50–200 mg/kg	Pacific white shrimp (*Penaeus vannamei*)	WSSV	Gholamhosseini et al. (2020b)
Geniposidic acid	–	50 mg/kg	Red swamp crayfish (*Procambarus clarkii*)	WSSV	Huang et al. (2020)
Chinese goldthread (*Coptis chinensis* Franch)	Berberine	30–50 mg/kg	Prussian carp (*Carassius gibelio*)	CyHV-2	Su et al. (2021)

Veterinarni Medicina, 68, 2023 (12): 449–463

https://doi.org/10.17221/96/2023-VETMED

Table S3 to be continued

Anti-bacteria					
Herbal name	Compound	Dose	Fish species	Function	References
Holy basil (*Ocimum Sanctum*)	Methanolic extract	100–200 mg/kg	Greasy grouper (*Epinephelus tauvina*)	Against *Vibrio harveyi* infection	Sivaram et al. (2004)
Guava (*Psidium guajava*)	Leaf extracts	1–10 mg/g	Nile tilapia (*Oreochromis niloticus*)	Enhance the resistance to *Aeromonas hydrophila*	Gobi et al. (2016)
Peppermint (*Mentha piperita*)	Extract	3%	Rainbow trout (*Oncorhynchus mykiss*)	Improve against *Yersinia ruckeri*	Adel et al. (2016)
Chinaberry tree (*Melia Azedarach*)	Extract	1.5 g/kg	Major south asian carp (*Catla catla*)	Against *Aeromonas hydrophila* infection	Rajeshwari et al. (2016)
Pumpkin (*Cucurbita mixta*)	–	4, 6 g/kg	Mozambique tilapia (*Oreochromis mossambicus*)	Enhance disease resistance against *Aeromonas hydrophila*	Musthafa et al. (2017)
Lead tree (*Leucaena leucocephala*)	Powder	33%	African sharp-tooth catfish (*Clarias gariepinus*)	Enhance against infection by *Vibrio harveyi*, *Pseudomonas aeruginosa*	Verma et al. (2018)
Velvet bean (*Mucuna pruriens*)	Powder	4–6 g/kg	Mozambique tilapia (*Oreochromis mossambicus*)	Against *Aeromonas hydrophila* infection	Musthafa et al. (2018)
Mongolian wild onion (*Allium mongolicum Regel*)	Flavonoids	40 mg/kg	Northern snakehead (*Channa argus*)	Increase disease resistance against *Aeromonas hydrophila*	Li et al. (2018)
Basil (*Ocimum gratissimum*)	Leaf extract	12 g/kg	African sharp-tooth catfish (*Clarias gariepinus*)	Enhance fish challenge against *Listeria monocytogenes*	Abdel-Tawwab et al. (2018)
Mangrove apple (*Sonneratia caseolaris*)	Extract	3.17 mg/ml	African sharp-tooth catfish (*Clarias gariepinus*)	Prevent against *Edwardsiella tarda* infection	Aznan et al. (2018)
Common purslane (*Portulaca oleracea* L.)	Leaf powder	1%–3%	Nile tilapia (*Oreochromis niloticus*)	Improve resistance against *Aeromonas hydrophila* infection	Abdel-Razek et al. (2019)

Veterinarni Medicina, 68, 2023 (12): 449–463
https://doi.org/10.17221/96/2023-VETMED

Table S3 to be continued

Anti-bacteria					
Herbal name	Compound	Dose	Fish species	Function	References
San-Huang-San	–	500 mg/kg	Pacific white shrimp (*Litopenaeus vannamei*)	Enhance resistance to *Vibrio parahaemolyticus*	Zhai and Li (2019)
Garlic (*Allium sativum*)	Extract	1.0 g	Nile tilapia (*Oreochromis niloticus*)	Prevent *Streptococcus iniae* infection	Foysal et al. (2019)
Smoketree (*Cotinus coggygria*)	Methanolic extract	24 mg/32.34 g body weight	Rainbow trout (*Oncorhynchus mykiss*)	Against *Aeromonas hydrophila* infection	Bilen and Elbeshti (2019)
Jojoba (*Simmondsia chinensis*)	Powder	0.5–2 g/kg	Nile tilapia (*Oreochromis niloticus*)	Against *Aeromonas hydrophila* infection	Sarhan et al. (2019)
Assam tea (*Camellia sinensis*)	Extract	2 g/kg	Nile tilapia (*Oreochromis niloticus*)	Offer resistance against *Streptococcus agalactiae* infection	Van Doan et al. (2019c)
Beard lichen (*Usnea barbata*)	Extract	4 mg/ 17.41 ± 0.3 g body weight/ day	Rainbow trout (*Oncorhynchus mykiss*)	Prevent against *Lactococcus garvieae* infection	Bilen et al. (2019)
Peppermint (*Mentha piperita*)	Essential oil	0.25%	Nile tilapia (*Oreochromis niloticus*)	Prevent against *Streptococcus agalactiae* infection	de Souza Silva et al. (2019)
Mix Fingerroot (*Boesenbergia pandurata*), Indian nightshade (*Solanum ferox*) and Pinecone ginger (*Zingiber zerumbet*)	Plant extracts	Fingerroot (*Boesenbergia pandurata*) 600 mg/l, Indian nightshade (*Solanum Ferox*) 900 mg/l, *Pinecone ginger* (*Zingiber zerumbet*) 200 mg/l	Nile tilapia (*Oreochromis niloticus*)	Enhance the resistance to *Aeromonas hydrophila* and *Pseudomonas* fluorescens	Hardi et al. (2019)
Maidenhair tree (*Ginkgo biloba*)	Leaf extract	10 g/kg	Common carp (*Cyprinus carpio*)	Improve survival rate of common carp after *Aeromonas hydrophila* infection	Bao et al. (2019)

Veterinarni Medicina, 68, 2023 (12): 449–463

https://doi.org/10.17221/96/2023-VETMED

Table S3 to be continued

Anti-bacteria					
Herbal name	Compound	Dose	Fish species	Function	References
Coriander (*Coriandrum sativum*)	Extract	2%	Rainbow trout (*Oncorhynchus mykiss*)	Improve resistance against *Yersinia ruckeri* infection	Naderi Farsani et al. (2019)
Prepared foxglove root (*Radix Rehmanniae Preparata*)	–	0.2%	Bulatmai barbel (*Luciobarbus capito*)	Enhance the disease resistance of *Aeromonas hydrophila*	Wu et al. (2019)
Lingzhi mushroom (*Ganoderma lucidum*) polysaccharides	–	2 g/kg	Giant river prawn (*Macrobrachium rosenbergii*)	Enhance the disease resistance of *Aeromonas hydrophila*	Mohan et al. (2019)
Gale of the wind (*Phyllanthus amarus*)	Extract	20 g/kg	Pacific white shrimp (*Litopenaeus vannamei*)	Enhance resistance to *Vibrio alginolyticus*	Ngo et al. (2020)
Moringa (*Moringa oleifera*)	Leave	40%	Gibel carp (*Carassius auratus gibelio*)	Increase disease resistance against *Aeromonas hydrophila* via TLR2 pathway	Zhang et al. (2020b)
Fern (*Adiantum capillus-veneris*)	Leave	2%	Common carp (*Cyprinus carpio*)	Enhance serum and mucosal bactericidal activities against different pathogenic bacteria	Hoseinifar et al. (2020a)
Tears of the virgin (*Eleutherine bulbosa*)	Powder	12.5 g/kg	Pacific white shrimp (*Litopenaeus vannamei*)	Enhance resistance against *Vibrio parahaemolyticus* infection	Munaeni et al. (2020)
Thumbai (*Leucas aspera*)	Powder	8 g/kg	Nile tilapia (*Oreochromis niloticus*)	Increase resistance against *Streptococcus* infection	Kurian et al. (2020)
Geniposide	–	100 mg/kg	Crucian carp (*Carassius carassius*)	Improve disease resistance against *Aeromonas hydrophila* infection	He et al. (2020)
Horse mint (*Mentha longifolia*)	Hydroalcoholic extract	0.2%	Rainbow trout (*Oncorhynchus mykiss*)	Improve resistance to bacterial disease	Heydari et al. (2020)

Veterinarni Medicina, 68, 2023 (12): 449–463
https://doi.org/10.17221/96/2023-VETMED

Table S3 to be continued

Anti-bacteria					
Herbal name	Compound	Dose	Fish species	Function	References
Berberine	–	1 g/kg	Nile tilapia (*Oreochromis niloticus*)	Boost disease resistance	Hoseinifar et al. (2020a)
Rosemary (*Salvia rosmarinus*)	Leaf powder	10 g/kg	Nile tilapia (*Oreochromis niloticus*)	Improve disease resistance of *Oreochromis niloticus*	Naiel et al. (2020)
Garlic (*Allium sativum*)	Ethanolic extract	200 mg/kg	Rainbow trout (*Oncorhynchus mykiss*)	Prevent fungal growth in the culturing centre	Amani Denji et al. (2020)
Black cumin (*Nigella sativa*)	Extract	7500 mg/kg	Pacific white shrimp (*Litopenaeus vannamei*)	Control vibriosis	Nur et al. (2020)
Mongolian milkvetch (*Astragalus membranaceus*)	–	100 mg/kg	Goldfish (*Carassius auratus*)	Improve disease resistance	Wu (2020)
Chinese rhubarb (*Rheum officinale*)	Extract	0.1, 1.0 g/kg	Orange-spotted grouper (*Epinephelus coioides*)	Against *Vibrio arahaemolyticus*, *Vibrio* vulnificus, *Vibrio alginolyticus*, *Vibrio carchariae*, *Aeromonas*, *Edwardsiella tarda*	Kuo et al. (2020)
Yucca (*Yucca filamentosa*)	Extract	0.1%	Nile tilapia (*Oreochromis niloticus*)	Increase disease resistance	Abeer and Naena (2020)
Tamarind (*Tamarindus indica* L.)	Extract	15 g/kg	Nile tilapia (*Oreochromis niloticus*)	Protect against *Aeromonas hydrophila* infection	Adeniyi et al. (2021)
Box myrtle (*Myrica esculenta*)	Ethanolic leaf extract	40 mg/l (immerse)	Rainbow trout (*Oncorhynchus mykiss*)	Increase haematological indices and non-specific immunological parameters	Bhat et al. (2021)
Marjoram (*Origanum majorana*)	Extract	200 mg/kg	Common carp (*Cyprinus carpio*)	Suppress fish mortality during *Aeromonas hydrophila*	Yousefi et al. (2021)

Veterinarni Medicina, 68, 2023 (12): 449–463

https://doi.org/10.17221/96/2023-VETMED

Table S3 to be continued

Anti-bacteria					
Herbal name	Compound	Dose	Fish species	Function	References
Common water hyacinth (*Eichhornia crassipes*)	Leave	2.5, 5%	Spotted snakehead (*Channa punctata*)	Enhance disease resistance against *Vibrio harveyi* infection	Verma et al. (2021)
Double-bladed sargassum (*Sargassum cristaefolium*)	Powder	10, 20 g/kg	Pacific white shrimp (*Litopenaeus vannamei*)	Inhibit *Vibrio* spp. in the intestine of *Litopenaeus vannamei*	Jahromi et al. (2021)
Grape seed	Extract	20, 30 g/kg	Common carp (*Cyprinus carpio*)	Enhance disease resistance against *Aeromonas hydrophila*	Mehrinakhi et al. (2021)
Greek juniper (*Juniperus excelsa*)	Extract	4, 8 mg/kg	Rainbow trout (*Oncorhynchus mykiss*)	Provides protection against *Yersinia ruckeri*	Bilen et al. (2021)

WSSV = White Spot Syndrome Virus; CyHV-3 = Cyprinid Herpesvirus 3; GCRV = Grass Carp Reovirus; SVCV = Spring Viraemia of Carp Virus; GIV = Grouper Iridovirus; CyHV-2 = Cyprinid Herpesvirus 2; TLR2 pathway = Toll-Like Receptor 2

Veterinarni Medicina, 68, 2023 (12): 449–463

https://doi.org/10.17221/96/2023-VETMED

Table S4. Use of medicinal plants as anti-parasite in aquaculture

Parasite	Herbal name	Compound	Fish species	Dosage & function	References
White spot disease (*Ichthyophthirius multifiliis*)	Garlic (*Allium sativum*) and Chamomile (*Matricaria chamomilla*)	Extract	Sailfin molly (*Poecilia latipinna*)	Bath with 0.1 g/l Garlic (*Allium sativum*) and 0.4 g/l Chamomile (*Matricaria chamomilla*)	Sahandi et al. (2012)
White spot disease (*Ichthyophthirius multifiliis*)	Chinese gall (*Galla chinensis*)	Ethanol extract	Channel catfish (*Ictalurus punctatus*)	Kill all theronts at concentrations of 2.5–20 mg/l; terminate reproduction of tomonts at 40 mg/l	Zhang et al. (2013)
White spot disease (*Ichthyophthirius multifiliis*)	Orange climber (*Toddalia asiatica*)	Chelerythrine, oroxylonine	Goldfish (*Carassius auratus*)	Two bioactive compounds: chelerythrine and chloroxylonine; the EC50 were 0.55 mg/l and 1.90 mg/l	Shan et al. (2014)
Gill fluke (*Dactylogyrus*) *Vastator*	Spurge (*Euphorbia fischeriana*)	Ethyl acetate extract	Goldfish (*Carassius auratus*)	The ethyl acetate extract could kill adult parasite at 10–20 mg/l	Zhang et al. (2014)
Gill fluke (*Dactylogyrus*)	Yam (*Dioscorea zingiberensis*)	Ethanol extract	Goldfish (*Carassius auratus*)	EC50 of extract was 3.427 mg/l	Jiang et al. (2014)
Gill fluke (*Dactylogyrus*)	Maidenhair tree (*Ginkgo biloba*)	Ethanol extract	Goldfish (*Carassius auratus*)	EC50 of extract was 3.544 mg/l	Jiang et al. (2014)
White spot disease (*Ichthyophthirius multifiliis*)	Babchi (*Psoralea corylifolia*)	Methanol extract	Goldfish (*Carassius auratus*)	Reduced the number of theronts released from tomonts at 2.5 mg/l	Song et al. (2015)
Salmon fluke (*Gyrodactylus*) *turnbulli*	Ginger (*Zingiber officinale*)	Ethanolic ginger extract	Guppy [*Poecilia reticulata* (Peters)]	Significantly reduced infection bathing in ethanolic ginger extract (i.e. 5 and 7.5 ng/l for 90 and 30 min respectively)	Levy et al. (2015)
Gill fluke (*Dactylogyrus*)	Yam (*Dioscorea zingiberensis* C.H.)	Gracillin	Goldfish (*Carassius auratus*)	Gracillin was the main active compound, which showed the highest synergy rate (71.4%) and increased anthelmintic efficacies	Luo et al. (2016)
White spot disease (*Ichthyophthirius multifiliis*)	English lavender (*Lavandula angustifolia*)	Linalyl acetate (45.44%) and linalool (35.94%)	Small-scaled pacu (*Piaractus mesopotamicus*)	EC was 114 µl/l *in vitro*	Valladao et al. (2016)
White spot disease (*Ichthyophthirius multifiliis*)	Peppermint (*Mentha piperita*)	d-menthol (44.57%), p-menthone (22.95%)	Small-scaled pacu (*Piaractus mesopotamicus*)	EC was 227 µl/l *in vitro*	Valladao et al. (2016)

https://doi.org/10.17221/96/2023-VETMED

Table S4 to be continued

Parasite	Herbal name	Compound	Fish species	Dosage & function	References
White spot disease (*Ichthyophthirius multifiliis*)	Tea tree (*Melaleuca alternifolia*)	1-terpinen-4-ol (44.60%), cterpinene (22.36%), cterpinene (11.14%)	Small-scaled pacu (*Piaractus mesopotamicus*)	EC was 57 µl/l *in vitro*	Valladao et al. (2016)
White spot disease (*Ichthyophthirius multifiliis*)	Sweet wormwood (*Artemisia annua*)	Powder	Goldfish (*Carassius auratus*)	20 g/kg feed protects fish against Ichthyophthirius multifiliis infection	Wu et al. 2017
Salmon fluke (*Gyrodactylus kobayashii*)	Plume poppy (*Macleaya cordata*)	Methanol extract	Goldfish (*Carassius auratus*)	EC50 and EC90 of methanol extract were 8.6 and 25.5 mg/l respectively	Zhou et al. (2017)
Gill fluke (*Dactylogyrus minutus*)	Rosemary (*Rosmarinus officinalis*)	Aqueous extract	Common carp (*Cyprinus carpio*)	60–100 ml aqueous extract/100 g feed	Zoral et al. (2017)
Monogeneans	Copaiba Rana (*Copaifera duckei*)	Oleoresin	Small-scaled pacu (*Piaractus mesopotamicus*)	50 mg/l is effective against monogeneans and did not affect the health of Piaractus mesopotamicus	da Costa et al. (2017)
White spot disease (*Ichthyophthirius multifiliis*)	Tea tree (*Melaleuca alternifolia*) essential oil	Tea tree (*Melaleuca alternifolia*) essential oil	Pale catfish (*Rhamdia quelen*)	Significant reduction in the number (94.87%)	Baldissera et al. (2017, 2018)
Monogenea (*Cichlidogyrus Tilapiae*)	Basil (*Ocimum gratissimum*)	Ethanol extract	Nile tilapia (*Oreochromis niloticus*)	320 mg/l can kill the monogenean	Meneses et al. (2018)
White spot disease (*Ichthyophthirius multifiliis*)	Ginger (*Zingiber officinale*)	6-dehydroshogaol, 6-dehydro-10-ingerol	Grass carp (*Ctenopharyngodon idella*)	Sharply decreased the number of theronts released from encysted tomonts	Fu et al. (2019)
Salmon fluke (*Gyrodactylus*) *kobayashii*	Whorled Honey Flower (*Paris polyphylla*)	Methanol extract	Goldfish (*Carassius auratus*)	Kill *Gyrodactylus kobayashii* at 20 mg/l, the TI reached 37.9	Zhou et al. (2020)
Gill fluke (*Dactylogyrus vastator*)	Tridax daisy (*Tridax procumbens*)	80% ethanol leaves extract	Nile tilapia (*Oreochromis niloticus*)	4–6 g/kg diet stimulated fish performance, intestinal morphometry, antioxidants, immunity responses and resistance to *Dactylogyrus vastator*	Adeshina et al. (2021)
White spot disease (*Ichthyophthirius multifiliis*)	Mixture of Bai Wei (*Cynanchum atratum*) and Shrubby sophora (*Sophora flavescens*)	Ethanol extracts	Grass carp (*Ctenopharyngodon idella*)	6 mg/l with continuous usage for 10 days cured the infected grass carp	Fu et al. (2021)

EC50 = Median Effective Concentration; EC = Effective Concentration; EC90 = 90% Effective Concentration; TI = Therapeutic Index

https://doi.org/10.17221/96/2023-VETMED

REFERENCES

Abbas WT, Abumourad IM, Mohamed LA, Abbas HH, Authman M, Soliman WS, Elgendy MY. The role of the dietary supplementation of fenugreek seeds in growth and immunity in Nile Tilapia with or without cadmium contamination. Jordan J Biol Sci. 2019 Dec;12(5):649-56.

Abd El-Gawad EA, El Asely AM, Soror EI, Abbass AA, Austin B. Effect of dietary Moringa oleifera leaf on the immune response and control of Aeromonas hydrophila infection in Nile tilapia (Oreochromis niloticus) fry. Aquac Int. 2020 Feb;28(1):389-402.

Abdel-Razek N, Awad SM, Abdel-Tawwab M. Effect of dietary purslane (Portulaca oleracea L.) leaves powder on growth, immunostimulation, and protection of Nile tilapia, Oreochromis niloticus against Aeromonas hydrophila infection. Fish Physiol Biochem. 2019 Dec;45(6):1907-17.

Abdel-Tawwab M, Adeshina I, Jenyo-Oni A, Ajani EK, Emikpe BO. Growth, physiological, antioxidants, and immune response of African catfish, Clarias gariepinus (B.), to dietary clove basil, Ocimum gratissimum, leaf extract and its susceptibility to Listeria monocytogenes infection. Fish Shellfish Immunol. 2018 Jul;78:346-54.

Abdel-Tawwab M, El-Araby DA. Immune and antioxidative effects of dietary licorice (Glycyrrhiza glabra L.) on performance of Nile tilapia, Oreochromis niloticus (L.) and its susceptibility to Aeromonas hydrophila infection. Aquaculture. 2021 Jan;530:735828.

Abeer EK, Naena NA. Yucca plant as treatment for Pseudomonas aeruginosa Infection in Nile tilapia farms with emphasis on its effect on growth performance. Alex J Vet Sci. 2020 Jul;66(1): 64-75.

Adel M, Amiri AA, Zorriehzahra J, Nematolahi A, Esteban MA. Effects of dietary peppermint (Mentha piperita) on growth performance, chemical body composition and hematological and immune parameters of fry Caspian white fish (Rutilus frisii kutum). Fish Shellfish Immunol. 2015Aug;45(2):841-7.

Adel M, Pourgholam R, Zorriehzahra J, Ghiasi M. Hemato -–Immunological and biochemical parameters, skin antibacterial activity, and survival in rainbow trout (Oncorhynchus mykiss) following the diet supplemented with Mentha piperita against Yersinia ruckeri. Fish Shellfish Immunol. 2016 Aug;55:267-73.

Adeniyi O, Emikpe B, Olaifa F, Ogunbanwo ST. Effects of dietary tamarind (Tamarindus indica L.) leaves extract on growth performance, nutrient utilization, gut physiology, and susceptibility to Aeromonas hydrophila infection in Nile tilapia (Oreochromis niloticus L.). Int Aquat Res. 2021 Mar;13(1):37-51.

Adeshina I, Abdel-Tawwab M, Tijjani ZA, Tiamiyu LO, Jahanbakhshi A. Dietary Tridax procumbens leaves extract stimulated growth, antioxidants, immunity, and resistance of Nile tilapia, Oreochromis niloticus, to monogenean parasitic infection. Aquaculture. 2021 Feb;532(1): 736047.

Adeshina I, Jenyo- Oni A, Emikpe BO, Ajani EK, Abdel- Tawwab M. Stimulatory effect of dietary clove, Eugenia caryophyllata, bud extract on growth performance, nutrient utilization, antioxidant capacity, and tolerance of African catfish, Clarias gariepinus (B.), to Aeromonas hydrophila infection. J World Aquac Soc. 2019 Aug;50(2):390-405.

Afzali S, Wong W. Effects of dietary supplementation of Sonneratia alba extract on immune protection and disease resistance in goldfish against Aphanomyces invadans. Trop Biomed. 2019 Mar;36(1):274-88.

Ahmadifar E, Hoseinifar SH, Adineh H, Moghadam MS, Dawood MA. Assessing the impact of purslane (Portulaca oleracea L.) on growth performance, anti-oxidative, and immune activities in grass carp (Ctenopharyngodon idella). Ann Anim Sci. 2020 Oct;20(4):1427-40.

Ahmadifar E, Sheikhzadeh N, Roshanaei K, Dargahi N, Faggio C. Can dietary ginger (Zingiber officinale) alter biochemical and immunological parameters and gene expression related to growth, immunity and antioxidant system in zebrafish (Danio rerio)? Aquaculture. 2019 Apr;507:341-8.

Ahmadniaye Motlagh H, Safari O, Selahvarzi Y, Baghalian A, Kia E. Non-specific immunity promotion in response to garlic extract supplemented diets in female Guppy (Poecilia reticulata). Fish Shellfish Immunol. 2020b Feb;97:96-9.

Al-Khalaifah HS, Khalil AA, Amer SA, Shalaby SI, Badr HA, Farag MF, Altohamy DE, Abdel Rahman AN. Effects of dietary doum palm fruit powder on growth, antioxidant capacity, immune response, and disease resistance of African catfish, Clarias gariepinus (B.). Animals. 2020 Aug; 10(8):1407.

Amani Denji K, Soltani M, Rajabi Islami H, Kamali A. The antifungal effect of Allium sativum and Artemisia sieberia extracts on hatching and survival of Oncorhynchus mykiss larvae. Iran J Fish Sci. 2020 Mar;19(2):669-80.

Angela C, Wang W, Lyu H, Zhou Y, Huang X. The effect of dietary supplementation of Astragalus membranaceus and Bupleurum chinense on the growth performance, immune-related enzyme activities and genes expression in white shrimp, Litopenaeus vannamei. Fish Shellfish Immunol. 2020 Dec;107(Pt A):379-84.

Ayoub HF, El Tantawy MM, Abdel-Latif HM. Influence of Moringa (Moringa oleifera) and Rosemary (Rosmarinus Officinalis), and Turmeric (Curcuma longa) on Immune

https://doi.org/10.17221/96/2023-VETMED

Parameters and Challenge of Nile tilapia to Aeromonas hydrophila. J Life Sci. 2019;16(4):8-15.

Aznan AS, Lee KL, Low CF, Iberahim NA, Ibrahim WNW, Musa N, Yeong YS, Musa N. Protective effect of apple mangrove Sonneratia caseolaris extract in Edwardsiella tarda-infected African catfish, Clarias gariepinus. Fish Shellfish Immunol. 2018 Jul;78:338-45.

Baldissera MD, Souza CF, Baldisserotto B. Melaleuca alternifolia essential oil prevents bioenergetics dysfunction in spleen of silver catfish naturally infected with Ichthyophthirius multifiliis. Microb Pathog. 2018 Oct;123:47-51.

Baldissera MD, Souza CF, Moreira KL, da Rocha MIU, da Veiga ML, Baldisserotto B. Melaleuca alternifolia essential oil prevents oxidative stress and ameliorates the antioxidant system in the liver of silver catfish (Rhamdia quelen) naturally infected with Ichthyophthirius multifiliis. Aquaculture. 2017 Nov;480:11-6.

Bao L, Chen Y, Li H, Zhang J, Wu P, Ye K, Ai H, Chu W. Dietary Ginkgo biloba leaf extract alters immune-related gene expression and disease resistance to Aeromonas hydrophila in common carp Cyprinus carpio. Fish Shellfish Immunol. 2019 Nov;94:810-8.

Beltran JMG, Silvera DG, Ruiz CE, Campo V, Chupani L, Faggio C, Esteban MA. Effects of dietary Origanum vulgare on gilthead seabream (Sparus aurata L.) immune and antioxidant status. Fish Shellfish Immunol. 2020 Apr;99:452-61.

Bhat RAH, Rehman S, Tandel RS, Dash P, Bhandari A, Ganie PA, Shah TK, Pant K, Yousuf DJ, Bhat IA, Chandra S, Mallik SK, Sarma D. Immunomodulatory and antimicrobial potential of ethanolic extract of Himalayan Myrica esculanta in Oncorhynchus mykiss: Molecular modelling with Aeromonas hydrophila functional proteins. Aquaculture. 2021 Feb;533:736213.

Bilen S, Altief TAS, Ozdemir KY, Salem MOA, Terzi E, Guney K. Effect of lemon balm (Melissa officinalis) extract on growth performance, digestive and antioxidant enzyme activities, and immune responses in rainbow trout (Oncorhynchus mykiss). Fish Physiol Biochem. 2020 Jan;46(1):471-81.

Bilen S, Elbeshti H. A new potential therapeutic remedy against Aeromonas hydrophila infection in rainbow trout (Oncorhynchus mykiss) using tetra, Cotinus coggygria. J Fish Dis. 2019 Oct;42(10):1369-81.

Bilen S, Ispir S, Kenanoglu ON, Tastan Y, Guney K, Terzi E. Effects of Greek juniper (Juniperus excelsa) extract on immune responses and disease resistance against Yersinia ruckeri in rainbow trout (Oncorhynchus mykiss). J Fish Dis. 2021 Jun;44(6):729-38.

Bilen S, Sirtiyah AMA, Terzi E. Therapeutic effects of beard lichen, Usnea barbata extract against Lactococcus garvieae infection in rainbow trout (Oncorhynchus mykiss). Fish Shellfish Immunol. 2019 Apr;87:401-9.

Buyukdeveci ME, Balcazar JL, Demirkale I, Dikel S. Effects of garlic-supplemented diet on growth performance and intestinal microbiota of rainbow trout (Oncorhynchus mykiss). Aquaculture. 2018;486:170-4.

Chelladurai G, Maran BAV. Dietary supplementation of mushroom extract enhances growth and antioxidant levels of Babylonia spirata (Mollusca: Gastropoda). Aquac Rep. 2019 Nov;15:100218.

Chen X, Hao K, Yu X, Huang A, Zhu B, Wang G-x, Ling F. Magnolol protects Ctenopharyngodon idella kidney cells from apoptosis induced by grass carp reovirus. Fish Shellfish Immunol. 2018 Mar;74:426-35.

Chirawithayaboon P, Areechon N, Meunpol O. Hepatopancreatic antioxidant enzyme activities and disease resistance of Pacific white shrimp (Litopenaeus vannamei) fed diet supplemented with garlic (Allium sativum) extract. Agr Nat Resour. 2020;54(4):377-86.

Cui Q, Zhao Z, Yuan C. Effects of astragalus polysaccharides on hemocytes phagocytosis and gene expression of immune-related factors in Eriocheir sinensis. Aquac Int. 2020;28(5):1787-96.

da Costa JC, Valladao GMR, Pala G, Gallani SU, Kotzent S, Crotti AEM, Fracarolli L, da Silva JJM, Pilarski F. Copaifera duckei oleoresin as a novel alternative for treatment of monogenean infections in pacu Piaractus mesopotamicus. Aquaculture. 2017 Mar;471:72-9.

Dang H, Zhang T, Yi F, Ye S, Liu J, Li Q, Li H, Li R. Enhancing the immune response in the sea cucumber Apostichopus japonicus by addition of Chinese herbs Houttuynia cordata Thunb as a food supplement. Aquac Fish. 2019 May;4(3):114-21.

Dawood MA, Gewaily MS, Monier MN, Younis EM, Van Doan H, Sewilam H. The regulatory roles of yucca extract on the growth rate, hepato-renal function, histopathological alterations, and immune-related genes in common carp exposed with acute ammonia stress. Aquaculture. 2021 Mar;534:736287.

de Assis RWS, Urbinati EC. Physiological activity of Aloe vera in pacu (Piaractus mesopotamicus) inoculated with Aeromonas hydrophila. Fish Physiol Biochem. 2020 Aug;46(4):1421-30.

de Souza Silva LT, de Padua Pereira U, de Oliveira HM, Brasil EM, Pereira SA, Chagas EC, Jesus GFA, Cardoso L, Mourino JLP, Martins ML. Hemato-immunological and zootechnical parameters of Nile tilapia fed essential oil of Mentha piperita after challenge with Streptococcus agalactiae. Aquaculture. 2019 May;506:205-11.

Devi G, Harikrishnan R, Paray BA, Al-Sadoon MK, Hoseinifar SH, Balasundaram C. Effects of aloe-emodin on

https://doi.org/10.17221/96/2023-VETMED

innate immunity, antioxidant and immune cytokines mechanisms in the head kidney leucocytes of Labeo rohita against Aphanomyces invadans. Fish Shellfish Immunol. 2019 Apr;87:669-78.

Diab AM, Al-Khefa BT, Khalafallah MM, Salah AS, Farrag FA, Dawood MAO. Dietary methanolic extract of fenugreek enhanced the growth, haematobiochemical, immune responses, and resistance against Aeromonas hydrophila in Nile Tilapia, Oreochromis niloticus. Aquac Res. 2023 Feb;2023(8):1-13.

El Asely A, Amin A, Abd El-Naby AS, Samir F, El-Ashram A, Dawood MA. Ziziphus mauritiana supplementation of Nile tilapia (Oreochromis niloticus) diet for improvement of immune response to Aeromonas hydrophila infection. Fish Physiol Biochem. 2020 Aug;46(4):1561-75.

Elbesthi RTA, Ozdemir KY, Tastan Y, Bilen S, Sonmez AY. Effects of ribwort plantain (Plantago lanceolata) extract on blood parameters, immune response, antioxidant enzyme activities, and growth performance in rainbow trout (Oncorhynchus mykiss). Fish Physiol Biochem. 2020 Aug; 46(4):1295-307.

Espinosa C, Beltran JMG, Messina CM, Esteban MA. Effect of Jasonia glutinosa on immune and oxidative status of gilthead seabream (Sparus aurata L.). Fish Shellfish Immunol. 2020 May;100:58-69.

Faheem M, Khaliq S, Mustafa N, Rani S, Lone KP. Dietary Moringa oleferia leaf meal induce growth, innate immunity and cytokine expression in grass carp, Ctenopharyngodon idella. Aquac Nutr. 2020 Apr;26(4):1164-72.

Fayed WM, Khalil RH, Sallam GR, Mansour AT, Elkhayat BK, Omar EA. Estimating the effective level of Yucca schidigera extract for improvement of the survival, haematological parameters, immunological responses and water quality of European seabass juveniles (Dicentrarchus labrax). Aquac Rep. 2019 Nov;15:100208.

Fierro-Coronado JA, Luna-Gonzalez A, Caceres-Martinez CJ, Ruiz-Verdugo CA, Escamilla-Montes R, Diarte-Plata G, Flores-Miranda MDC, Alvarez-Ruiz P, Peraza-Gomez V. Effect of medicinal plants on the survival of white shrimp (Penaeus vannamei) challenged with WSSV and Vibrio parahaemolyticus. Lat Am J Aquat Res. 2019;47(2):377-81.

Foysal MJ, Alam M, Momtaz F, Chaklader MR, Siddik MA, Cole A, Fotedar R, Rahman M.M. Dietary supplementation of garlic (Allium sativum) modulates gut microbiota and health status of tilapia (Oreochromis niloticus) against Streptococcus iniae infection. Aquac Res. 2019 May;50(8):2107-16.

Fu YW, Guo SQ, Luo JJ, Sang CG, Lin DJ, Liu YM, Zhang QZ. Effectiveness assessment of plant mixtures against Ichthyophthirius multifiliis in grass carp Ctenopharyngodon idella. Aquaculture. 2021 Jan;530:735742.

Fu YW, Wang B, Zhang QZ, Xu DH, Liu YM, Hou TL, Guo SQ. Efficacy and antiparasitic mechanism of 10-gingerol isolated from ginger Zingiber officinale against Ichthyophthirius multifiliis in grass carp. Vet Parasitol. 2019 Jan;265:74-84.

Garcia Beltran JM, Espinosa C, Guardiola FA, Manuguerra S, Santulli A, Messina CM, Esteban MA. Effects of dietary dehydrated lemon peel on some biochemical markers related to general metabolism, welfare and stress in gilthead seabream (Sparus aurata L.). Aquac Res. 2019;50(11): 3181-91.

Gholamhosseini A, Adel M, Dawood MA, Banaee M. The potential benefits of Mentha longifolia on growth performance and innate immunity parameters in Caspian kutum (Rutilus frisii kutum). Aquac Res. 2020a;51(12): 5212-27.

Gholamhosseini A, Hosseinzadeh S, Soltanian S, Banaee M, Sureda A, Rakhshaninejad M, Heidari AA, Anbazpour H. Effect of dietary supplements of Artemisia dracunculus extract on the haemato- immunological and biochemical response, and growth performance of the rainbow trout (Oncorhynchus mykiss). Aquac Res. 2021;52(5):2097-109.

Gholamhosseini A, Kheirandish MR, Shiry N, Akhlaghi M, Soltanian S, Roshanpour H, Banaee M. Use of a methanolic olive leaf extract (Olea europaea) against white spot virus syndrome in Penaeus vannamei: Comparing the biochemical, hematological and immunological changes. Aquaculture. 2020b Nov;528:735556.

Gholipour Kanani H, Nobahar Z, Kakoolaki S, Jafarian H. Effect of ginger-and garlic-supplemented diet on growth performance, some hematological parameters and immune responses in juvenile Huso huso. Fish Physiol Biochem. 2014 Apr;40(2):481-90.

Giri SS, Jai Suda S, Sukumaran V, Park SC. Dietary emodin affects the growth performance, immune responses, and disease resistance of Labeo rohita against Aeromonas hydrophila. Aquac Int. 2016;24(1):85-99.

Giri SS, Jun JW, Sukumaran V, Park SC. Evaluation of dietary Hybanthus enneaspermus (Linn F. Muell.) as a growth and haemato-immunological modulator in Labeo rohita. Fish Shellfish Immunol. 2017 Sep;68:310-7.

Gobi N, Ramya C, Vaseeharan B, Malaikozhundan B, Vijayakumar S, Murugan K, Benelli G. Oreochromis mossambicus diet supplementation with Psidium guajava leaf extracts enhance growth, immune, antioxidant response and resistance to Aeromonas hydrophila. Fish Shellfish immunol. 2016 Nov;58:572-83.

Haetrakul T, Dunbar SG, Chansue N. Antiviral activities of Clinacanthus nutans (Burm.f.) Lindau extract against Cyprinid herpesvirus 3 in koi (Cyprinus carpio koi). J Fish Dis. 2018 Apr;41(4):581-7.

https://doi.org/10.17221/96/2023-VETMED

Hajirezaee S, Rafieepour A, Shafiei S, Rahimi R. Immunostimulating effects of Ginkgo biloba extract against toxicity induced by organophosphate pesticide, diazinon in rainbow trout, Oncorhynchus mykiss: innate immunity components and immune-related genes. Environ Sci Pollut Res. 2019 Mar;26(9):8798-807.

Hamed HS, Ismal SM, Faggio C. Effect of allicin on antioxidant defense system, and immune response after carbofuran exposure in Nile tilapia, Oreochromis niloticus. Comp Biochem Physiol Part C. 2021 Feb;240:108919.

Hardi E, Nugroho R, Kusuma I, Apriza A. Immunomodulatory effect and disease resistance from concoction three of Borneo plant extracts in tilapia, Oreochromis niloticus. J Aquac Indones. 2019;20(1):41-7.

Hassaan MS, Mohammady EY, Soaudy MR, El-Garhy HA, Moustafa MM, Mohamed SA, El-Haroun ER. Effect of Silybum marianum seeds as a feed additive on growth performance, serum biochemical indices, antioxidant status, and gene expression of Nile tilapia, Oreochromis niloticus (L.) fingerlings. Aquaculture. 2019 Jul;509: 178-87.

He G, Sun H, Liao R, Wei Y, Zhang T, Chen Y, Lin S. Effects of herbal extracts (Foeniculum vulgare and Artemisia annua) on growth, liver antioxidant capacity, intestinal morphology and microorganism of juvenile largemouth bass, Micropterus salmoides. Aquac Rep. 2022 Apr;23(7): 101081.

He M, Liu G, Liu Y, Yang K, Qi X, Huang A, Liu T, Wang G, Wang E. Effects of geniposide as immunostimulant on the innate immune response and disease resistance in crucian carp. Aquaculture. 2020 Dec;529:735713.

Heydari M, Firouzbakhsh F, Paknejad H. Effects of Mentha longifolia extract on some blood and immune parameters, and disease resistance against yersiniosis in rainbow trout. Aquaculture. 2020 Jan2020;515:734586.

Hoseinifar SH, Jahazi MA, Mohseni R, Raeisi M, Bayani M, Mazandarani M, Yousefi M, Van Doan H, Mozanzadeh MT. Effects of dietary fern (Adiantum capillus-veneris) leaves powder on serum and mucus antioxidant defence, immunological responses, antimicrobial activity and growth performance of common carp (Cyprinus carpio) juveniles. Fish Shellfish Immunol. 2020a Nov;106:959-66.

Hoseinifar SH, Shakouri M, Doan HV, Shafiei S, Yousefi M, Raeisi M, Yousefi S, Harikrishnan R, Reverter M. Dietary supplementation of lemon verbena (Aloysia citrodora) improved immunity, immune-related genes expression and antioxidant enzymes in rainbow trout (Oncorhynchus mykiss). Fish Shellfish Immunol. 2020b Apr;99: 379-85.

Hoseinifar SH, Shakouri M, Yousefi S, Van Doan H, Shafiei S, Yousefi M, Mazandarani M, Mozanzadeh MT, Tulino MG, Faggio C. Humoral and skin mucosal immune parameters, intestinal immune related genes expression and antioxidant defense in rainbow trout (Oncorhynchus mykiss) fed olive (Olea europea L.) waste. Fish Shellfish Immunol. 2020c May;100:171-8.

Hoseinifar SH, Sohrabi A, Paknejad H, Jafari V, Paolucci M, Van Doan H. Enrichment of common carp (Cyprinus carpio) fingerlings diet with Psidium guajava: the effects on cutaneous mucosal and serum immune parameters and immune related genes expression. Fish Shellfish Immunol. 2019 Mar;86:688-94.

Huang AG, Tan XP, Cui HB, Qi XZ, Zhu B, Wang GX. Antiviral activity of geniposidic acid against white spot syndrome virus replication in red swamp crayfish Procambarus clarkii. Aquaculture. 2020 Nov;528:735533.

Huang AG, Tan XP, Qu SY, Wang GX, Zhu B. Evaluation on the antiviral activity of genipin against white spot syndrome virus in crayfish. Fish Shellfish Immunol. 2019b Oct;93:380-6.

Huang AG, Tu X, Qi XZ, Ling F, Zhu B, Wang GX. Gardenia jasminoides Ellis inhibit white spot syndrome virus replication in red swamp crayfish Procambarus clarkii. Aquaculture. 2019a Apr;504:239-47.

Ibrahim RE, El-Houseiny W, Behairy A, Mansour MF, Abd-Elhakim YM. Ameliorative effects of Moringa oleifera seeds and leaves on chlorpyrifos-induced growth retardation, immune suppression, oxidative stress, and DNA damage in Oreochromis niloticus. Aquaculture. 2019 Apr;505:225-34.

Jafarinejad R, Gharaei A, Mirdar Harijani J. Dietary ginger improve growth performance, blood parameters, antioxidant capacity and gene expression in Cyprinus carpio. Iran J Fish Sci. 2020;19(3):1237-52.

Jahromi ST, Pourmozaffar S, Jahanbakhshi A, Rameshi H, Gozari M, Khodadadi M, Sohrabipour J, Behzadi S, Barzkar N, Nahavandi R, Zahedi MR, Moezzi M. Effect of different levels of dietary Sargassum cristaefolium on growth performance, hematological parameters, histological structure of hepatopancreas and intestinal microbiota of Litopenaeus vannamei. Aquaculture. 2021 Feb; 533:736130.

Jia R, Gu Z, He Q, Du J, Cao L, Jeney G, Xu P, Yin G. Antioxidative, anti-inflammatory and hepatoprotective effects of Radix Bupleuri extract against oxidative damage in tilapia (Oreochromis niloticus) via Nrf2 and TLRs signaling pathway. Fish Shellfish Immunol. 2019 Oct;93: 395-405.

Jiang C, Wu ZQ, Liu L, Liu GL, Wang GX. Synergy of herbal ingredients combination against Dactylogyrus spp. in an infected goldfish model for monogenean management. Aquaculture. 2014 Sep;433:115-8.

https://doi.org/10.17221/96/2023-VETMED

Kaleo IV, Gao Q, Liu B, Sun C, Zhou Q, Zhang H, Shan F, Xiong Z, Bo L, Song C. Effects of Moringa oleifera leaf extract on growth performance, physiological and immune response, and related immune gene expression of Macrobrachium rosenbergii with Vibrio anguillarum and ammonia stress. Fish Shellfish Immunol. 2019 Jun;89:603-13.

Kannan B, Felix N, Panigrahi A, Ahilan, B. Herbal extracts modulate growth, immune responses and resistance to Aeromonas hydrophila infection in GIFT tilapia (Oreochromis niloticus). Aquac Res. 2022 Sep;53(13):4627-37.

Karatas T, Korkmaz F, Karatas A, Yildirim S. Effects of Rosemary (Rosmarinus officinalis) extract on growth, blood biochemistry, immunity, antioxidant, digestive enzymes and liver histopathology of rainbow trout, Oncorhynchus mykiss. Aquac Nutr. 2020;26(5):1533-41.

Karimi Pashaki A, Ghasemi M, Zorriehzahra M, Shrif Rohani M, Hosseini S. Effects of dietary garlic (Allium sativum) extract on survival rate, blood and immune parameters changes and disease resistance of Common carp (Cyprinus carpio carpio Linnaeus, 1758) against Spring Viremia of carp (SVC). Iran J Fish Sci. 2020;19(3): 1024-39.

Kuo IP, Lee PT, Nan FH. Rheum officinale extract promotes the innate immunity of orange-spotted grouper (Epinephelus coioides) and exerts strong bactericidal activity against six aquatic pathogens. Fish Shellfish Immunol. 2020 Jul;102:117-24.

Kurian A, Van Doan H, Tapingkae W, Elumalai P. Modulation of mucosal parameters, innate immunity, growth and resistance against Streptococcus agalactiae by enrichment of Nile tilapia (Oreochromis niloticus) diet with Leucas aspera. Fish Shellfish Immunol. 2020 Feb;97:165-72.

Lamichhane S, Khanal M, Labh S. Effect of Dietary Aloe vera extract on survival, growth and hepato-somatic index (HSI) of common carp Cyprinus Carpio (Linnaeus, 1758). Jr Aqua Mar Bio Eco: JAMBE-104. 2020;4:1.72-0.041.

Levy G, Zilberg D, Paladini G, Fridman S. Efficacy of ginger-based treatments against infection with Gyrodactylus turnbulli in the guppy (Poecilia reticulata (Peters)). Vet Parasitol. 2015 Apr;209(3-4):235-41.

Li H, Qiang J, Song C, Xu P. Transcriptome profiling reveal Acanthopanax senticosus improves growth performance, immunity and antioxidant capacity by regulating lipid metabolism in GIFT (Oreochromis niloticus). Comp Biochem Physiol Part D. 2021 Mar;37:100784.

Li H, Wu M, Jiang J, Sun X, Chen L, Feng M, Yuan D, Wen Z, Qin C.The extracts of Angelica sinensis restore the digestive and absorptive capacity through improving antioxidant status in digestive organs of fish treated with trichlorfon. Aquac Res. 2019a Feb;50(2):490-504.

Li H, Yang D, Li Z, He M, Li F, Jiang J, Tang S, Peng P, Du W, Ma Y, Liu Y. Effects of Angelica sinensis extracts on lipid oxidation in fish feeds and growth performance of juvenile Jian carp (Cyprinus carpio var. Jian). Anim Nutr. 2019b Mar;5(1):109-14.

Li M, Zhu X, Tian J, Liu M, Wang G. Dietary flavonoids from Allium mongolicum Regel promotes growth, improves immune, antioxidant status, immune-related signaling molecules and disease resistance in juvenile northern snakehead fish (Channa argus). Aquaculture. 2018 Feb;501:473-81.

Liu B, Ge X, He Y, Xie J, Xu P, He Y, Zhou Q, Pan L, Chen R. Effects of anthraquinones extracted from Rheum officinale Bail on the growth, non-specific immune response of Macrobrachium rosenbergii. Aquaculture. 2010 Dec; 310(1-2):13-9.

Liu M, Yu Q, Xiao H, Yi Y, Cheng H, Putra DF, Huang Y, Zhang Q, Li P. Antiviral activity of Illicium verum Hook. f. extracts against grouper iridovirus infection. J Fish Dis. 2020 May;43(5):531-40.

Luo F, Ling F, Geng T, Liu G, Jiang C, Wang G. Systematic screening identifies synergistic combinations of traditional Chinese medicines and ingredients against Dactylogyrus infections using a goldfish model. Aquaculture. 2016 Jun;459:198-202.

Maldonado-Garcia M, Angulo C, Vazquez-Martinez J, Sanchez V, Lopez MG, Reyes-Becerril M. Antioxidant and immunostimulant potentials of Chenopodium ambrosioides L. in Pacific red snapper (Lutjanus peru). Aquaculture. 2019 Nov;513:734414.

Mehrabi Z, Firouzbakhsh F, Rahimi-Mianji G, Paknejad H. Immunostimulatory effect of Aloe vera (Aloe barbadensis) on non-specific immune response, immune gene expression, and experimental challenge with Saprolegnia parasitica in rainbow trout (Oncorhynchus mykiss). Aquaculture. 2019 Mar;503:330-8.

Mehrabi Z, Firouzbakhsh F, Rahimi-Mianji G, Paknejad H. Immunity and growth improvement of rainbow trout (Oncorhynchus mykiss) fed dietary nettle (Urtica dioica) against experimental challenge with Saprolegnia parasitica. Fish Shellfish Immunol. 2020 Sep;104(12):74-82.

Mehrinakhi Z, Ahmadifar E, Sheikhzadeh N, Moghadam MS, Dawood MAO. Extract of grape seed enhances the growth performance, humoral and mucosal immunity, and resistance of common carp (Cyprinus carpio) against Aeromonas hydrophila. Ann Anim Sci. 2021 Jan;21(1): 217-32.

Meneses J, do Couto M, Sousa N, Cunha FdS, Abe H, Ramos F, Chagas EC, Chaves FCM, Martins ML, Maria AN, Carneiro PCF, Fujimoto RY. Efficacy of Ocimum gratissimum essential oil against the monogenean Cichlido-

https://doi.org/10.17221/96/2023-VETMED

gyrus tilapiae gill parasite of Nile tilapia. Arq Bras Med Vet Zootec. 2018 Mar;70(2):497-504.

Mohan K, Muralisankar T, Uthayakumar V, Chandirasekar R, Rajan DK. Dietary Ganoderma lucidum polysaccharides to enhance the growth, immune response and disease resistance of freshwater prawn Macrobrachium rosenbergii. Aquac Rep. 2019 Jul;14:100203.

Moustafa EM, Dawood MA, Assar DH, Omar AA, Elbialy ZI, Farrag FA, Shukry M, Zayed MM. Modulatory effects of fenugreek seeds powder on the histopathology, oxidative status, and immune related gene expression in Nile tilapia (Oreochromis niloticus) infected with Aeromonas hydrophila. Aquaculture. 2020 Jan;515:734589.

Munaeni W, Widanarni, Yuhana M, Setiawati M, Wahyudi AT. Effect in white shrimp Litopenaeus vannamei of Eleutherine bulbosa (Mill.) Urb. Powder on immune genes expression and resistance against Vibrio parahaemolyticus infection. Fish Shellfish Immunol. 2020 Jul; 102:218-27.

Musthafa MS, Ali ARJ, Kumar MSA, Paray BA, Al-Sadoon MK, Balasundaram C, Harikrishnan R. Effect of Cucurbita mixta (L.) seed meal enrichment diet on growth, immune response and disease resistance in Oreochromis mossambicus. Fish Shellfish Immunol. 2017 Sep;68: 509-15.

Musthafa MS, Asgari SM, Kurian A, Elumalai P, Ali ARJ, Paray BA, Al-Sadoon MK. Protective efficacy of Mucuna pruriens (L.) seed meal enriched diet on growth performance, innate immunity, and disease resistance in Oreochromis mossambicus against Aeromonas hydrophila. Fish Shellfish Immunol. 2018 Apr;75:374-80.

Naderi Farsani M, Hoseinifar SH, Rashidian G, Ghafari Farsani H, Ashouri G, Van Doan H. Dietary effects of Coriandrum sativum extract on growth performance, physiological and innate immune responses and resistance of rainbow trout (Oncorhynchus mykiss) against Yersinia ruckeri. Fish Shellfish Immunol. 2019 Aug;91: 233-40.

Naiel MA, Ismael NE, Shehata SA. Ameliorative effect of diets supplemented with rosemary (Rosmarinus officinalis) on aflatoxin B1 toxicity in terms of the performance, liver histopathology, immunity and antioxidant activity of Nile Tilapia (Oreochromis niloticus). Aquaculture. 2019 Sep;511:734264.

Naiel MAE, Ismael NEM, Negm SS, Ayyat MS, Al- Sagheer AA. Rosemary leaf powder-supplemented diet enhances performance, antioxidant properties, immune status, and resistance against bacterial diseases in Nile Tilapia (Oreochromis niloticus). Aquaculture. 2020 Sep;526:735370.

Naqi JA, Mateen A, Hussain D, Tahir HM, Hussain S, Tabasum A. Effect of Allium sativum supplemented diets on growth and haematological responses in Nile Tilapia (Oreochromis niloticus). Pak J Zool. 2019 Feb;51(1):257-63.

Ngo HV, Huang HT, Lee PT, Liao ZH, Chen HY, Nan FH. Effects of Phyllanthus amarus extract on nonspecific immune responses, growth, and resistance to Vibrio alginolyticus in white shrimp Litopenaeus vannamei. Fish Shellfish Immunol. 2020 Dec;107:1-8.

Nur I, Munaeni W, Abidin LOB. Assessment of antibacterial and immunostimulating activity of black cumin (Nigella sativa) extract against vibriosis in white shrimp (Litopenaeus vannamei). Thai J Vet Med. 2020 Dec;50(4):549-57.

Ojha ML, Chadha NK, Saini VP, Damroy S, Gupta CP, Savant PB. Effect of ethanolic extract of Pedalium murex on growth and haemato-immunological parameters of Labeo rohita. Proc Natl. Acad Sci India Sect B Biol Sci. 2014;84:997-1003.

Paknejad H, Hosseini Shekarabi SP, Shamsaie Mehrgan M, Hajimoradloo A, Khorshidi Z, Rastegari S. Dietary peppermint (Mentha piperita) powder affects growth performance, hematological indices, skin mucosal immune parameters, and expression of growth and stress-related genes in Caspian roach (Rutilus caspicus). Fish Physiol Biochem. 2020 Oct;46(5):1883-95.

Palanikumar P, Daffni Benitta DJ, Lelin C, Thirumalaikumar E, Michaelbabu M, Citarasu T. Effect of Argemone mexicana active principles on inhibiting viral multiplication and stimulating immune system in Pacific white leg shrimp Litopenaeus vannamei against white spot syndrome virus. Fish Shellfish Immunol. 2018 Apr;75:243-52.

Paray BA, Hoseini SM, Hoseinifar SH, Van Doan H. Effects of dietary oak (Quercus castaneifolia) leaf extract on growth, antioxidant, and immune characteristics and responses to crowding stress in common carp (Cyprinus carpio). Aquaculture. 2020 Jul;524:735276.

Qian X, Zhu F. Hesperetin protects crayfish Procambarus clarkii against white spot syndrome virus infection. Fish Shellfish Immunol. 2019 Oct;93:116-23.

Rahman ANA, ElHady M, Hassanin ME, Mohamed AA-R. Alleviative effects of dietary Indian lotus leaves on heavy metals-induced hepato-renal toxicity, oxidative stress, and histopathological alterations in Nile tilapia, Oreochromis niloticus (L.). Aquaculture. 2019 Jul;509:198-208.

Rajabiesterabadi H, Hoseini SM, Fazelan Z, Hoseinifar SH, Doan HV. Effects of dietary turmeric administration on stress, immune, antioxidant and inflammatory responses of common carp (Cyprinus carpio) during copper exposure. Aquac Nutr. 2020;26(4):1143-53.

Rajeshwari S, Rajan M, Pavaraj M, Sevarkodiyone S. Effect of Melia azedarach extract on some selected physiological parameters of (Catla catla). Int J Aquac Fish Sci. 2016 Jul;2(1):27-30.

https://doi.org/10.17221/96/2023-VETMED

Ramezanzadeh S, Abedian Kenari A, Esmaeili MA. Immunohematological parameters of rainbow trout (Oncorhynchus mykiss) fed supplemented diet with different forms of barberry root (Berberis vulgaris). Comp Clin Path. 2019;29:177-87.

Rashidian G, Kajbaf K, Prokic MD, Faggio C. Extract of common mallow (Malvae sylvestris) enhances growth, immunity, and resistance of rainbow trout (Oncorhynchus mykiss) fingerlings against Yersinia ruckeri infection. Fish Shellfish Immunol. 2020 Jan;96:254-61.

Rufchaei R, Mirvaghefi A, Hoseinifar SH, Valipour A, Nedaei S. Effects of dietary administration of water hyacinth (Eichhornia crassipes) leaves extracts on innate immune parameters, antioxidant defence and disease resistance in rainbow trout (Oncorhynchus mykiss). Aquaculture. 2020 Jan;515:734533.

Safari O, Sarkheil M, Paolucci M. Dietary administration of ferula (Ferula asafoetida) powder as a feed additive in diet of koi carp, Cyprinus carpio koi: effects on hemato-immunological parameters, mucosal antibacterial activity, digestive enzymes, and growth performance. Fish Physiol Biochem. 2019 Aug;45(4):1277-88.

Sahandi J, Kanani H, Asgarabad F. Influence of garlic (Allium sativum) and mother worth (Matricaria chamomilla) extract effects on Ichthyophtirius multifilus parasite treatment in sail fin molly (Poecilia latipinna) ornamental fish. Glob Vet. 2012;9(3):362-6.

Salomon R, Firmino JP, Reyes-Lopez FE, Andree KB, Gonzalez-Silvera D, Esteban MA, Tort L, Quintela JC, Pinilla-Rosas JM, Vallejos-Vidal E, Gisbert E. The growth promoting and immunomodulatory effects of a medicinal plant leaf extract obtained from Salvia officinalis and Lippia citriodora in gilthead seabream (Sparus aurata). Aquaculture. 2020 Jul;524:735291.

Sanchez CJG, Velasco RR, Doctolero JS. Young turmeric (Curcuma longa) tuber as feed additive for the growth and survival of Nile tilapia (Oreochromis niloticus L.). Int J Fish Aquat Stud. 2019;7(6):181-4.

Sarhan IA, Abdel-Aziz SA, Said AA, Abdel-Aleim A-AF, Awad SM. Effect of dietary supplementation of extracted jojoba meal on hematology , biochemical parameters and disease resistance in Nile tilapia (Oreochromis niloticus) infected by Aeromonas hydrophila. Egypt J Aquac. 2019 Sep;9(3):13-31.

Shan XF, Meng QF, Kang YH, Bian Y, Gao YH, Wang WL, Ai-dong Q. Isolation of active compounds from methanol extracts of Toddalia asiatica against Ichthyophthirius multifiliis in goldfish (Carassius auratus). Vet Parasitol. 2014 Jan;199(3-4):250-4.

Sharma J, Kumar N, Singh SP, Singh A, Harikrishna V. Evaluation of immunostimulatory properties of prickly chaff flower Achyranthes aspera in rohu Labeo rohita fry in pond conditions. Aquaculture. 2019 Apr;505:183-9.

Shen YF, Hu Y, Zhang Z, Liu L, Chen C, Tu X, Wang GX, Zhu B. Saikosaponin D efficiently inhibits SVCV infection in vitro and in vivo. Aquaculture. 2019 Apr;504:281-90.

Shen YF, Liu L, Feng C-Z, Hu Y, Chen C, Wang GX, Zhu B. Synthesis and antiviral activity of a new coumarin derivative against spring viraemia of carp virus. Fish Shellfish Immunol. 2018 Oct;81:57-66.

Shi F, Lu Z, Yang M, Li F, Zhan F, Zhao L, Li Y, Li Q, Li J, Li J, Lin L, Qin Z. Astragalus polysaccharides mediate the immune response and intestinal microbiota in grass carp (Ctenopharyngodon idellus). Aquaculture. 2021 Mar;534:736205.

Sikotariya S. Effect of Allium cepa (Onion) powder on the growth and survival in Cirrhinus mrigala fingerlings. Int J Pure Appl Biosci. 2019;7(5):186-96.

Sirakov I, Velichkova K, Stoyanova S, Zhelyazkov G, Staykov Y. The effect of diet supplemented with dandelion's (Taraxacum Officinale) extract on the productive and blood parameters of common carp (Cyprinus Carpio L.), cultivated in the recirculation system. Maced Vet Rev. 2019;42:131-39.

Sivaram V, Babu M, Immanuel G, Murugadass S, Citarasu T, Marian MP. Growth and immune response of juvenile greasy groupers (Epinephelus tauvina) fed with herbal antibacterial active principle supplemented diets against Vibrio harveyi infections. Aquaculture. 2004 Aug;237 (1-4):9-20.

Soares MP, Cardoso IL, Ishikawa MM, de Oliveira A, Sartoratto A, Jonsson CM, de Queiroz SCDN, Duarte MCT, Rantin FT, Sampaio FG. Effects of Artemisia annua alcohol extract on physiological and innate immunity of Nile tilapia (Oreochromis niloticus) to improve health status. Fish Shellfish Immunol. 2020 Oct;105:369-77.

Song C, Liu B, Jiang S, Xiong Y, Sun C, Zhou Q, Jiang Z, Liu B, Zhang H. Anthraquinone extract from Rheum officinale Bail improves growth performance and Toll-Relish signaling-regulated immunity and hyperthermia tolerance in freshwater prawn Macrobrachium nipponense. 3 Biotech. 2020 Dec;10(12):526.

Song K, Ling F, Huang A, Dong W, Liu G, Jiang C, Zhang Q, Wang G. In vitro and in vivo assessment of the effect of antiprotozoal compounds isolated from Psoralea corylifolia against Ichthyophthirius multifiliis in fish. Int J Parasitol Drugs Drug Resist. 2015 Aug;5(2):58-64.

Song X, Feng Z, Zhang Y, Zhu W. Regulation of dietary astragalus polysaccharide (APS) supplementation on the non-specific immune response and intestinal microbiota of sea cucumber Apostichopus japonicus. Fish Shellfish Immunol. 2019 Nov;94:517-24.

https://doi.org/10.17221/96/2023-VETMED

Srichaiyo N, Tongsiri S, Hoseinifar SH, Dawood MAO, Esteban M, Ringø E, Van Doan H. The effect of fishwort (Houttuynia cordata) on skin mucosal, serum immunities, and growth performance of Nile tilapia. Fish Shellfish Immunol. 2020a Mar;98:193-200.

Srichaiyo N, Tongsiri S, Hoseinifar SH, Dawood MAO, Jaturasitha S, Esteban MA, Ringø E, Van Doan H. The effects gotu kola (Centella asiatica) powder on growth performance, skin mucus, and serum immunity of Nile tilapia (Oreochromis niloticus) fingerlings. Aquac Rep. 2020b Mar;16:100239.

Su M, Tang R, Wang H, Lu L. Suppression effect of plant-derived berberine on cyprinid herpesvirus 2 proliferation and its pharmacokinetics in Crucian carp (Carassius auratus gibelio). Antiviral Res. 2021 Feb;186:105000.

Sukumaran V, Park SC, Giri SS. Role of dietary ginger Zingiber officinale in improving growth performances and immune functions of Labeo rohita fingerlings. Fish Shellfish Immunol. 2016 Oct;57:362-70.

Sun Z, Chen L, Liu Q, Mai K, Xu M, Zhou Y, Su N, Ye C. Effects of dietary Senecio scandens buch-ham extracts on growth performance, plasma biochemical, histology and the expression of immune-related genes in hybrid grouper (Epinephelus lanceolatus♂ × Epinephelus fuscoguttatus♀). Fish Shellfish Immunol. 2020 Mar;98:681-90.

Ta I, Sa D, Ha A. Growth performance and hematological indices of Clarias gariepinus (Burchel, 1822) fingerlings fed varying levels of Telfairia occidentalis leaf meal additives. Int J Fish Aquat Stud. 2019;7(5):442-5.

Tan X, Sun Z. Dietary dandelion extract improved growth performance, immunity, intestinal morphology and microbiota composition of golden pompano Trachinotus ovatus. Aquac Rep. 2020 Nov;18:100491.

Tan X, Sun Z, Ye C, Lin H. The effects of dietary Lycium barbarum extract on growth performance, liver health and immune related genes expression in hybrid grouper (Epinephelus lanceolatus♂ × E. fuscoguttatus♀) fed high lipid diets. Fish Shellfish Immunol. 2019 Apr;87:847-52.

Valladao GM, Gallani SU, Ikefuti CV, da Cruz C, Levy-Pereira N, Rodrigues MV, Pilarski F. Essential oils to control ichthyophthiriasis in pacu, Piaractus mesopotamicus (Holmberg): special emphasis on treatment with Melaleuca alternifolia. J Fish Dis. 2016 Oct;39(10):1143-52.

Van Doan H, Hoseinifar SH, Chitmanat C, Jaturasitha S, Paolucci M, Ashouri G, Dawood MAO, Esteban MA. The effects of Thai ginseng, Boesenbergia rotunda powder on mucosal and serum immunity, disease resistance, and growth performance of Nile tilapia (Oreochromis niloticus) fingerlings. Aquaculture. 2019a Nov;513:734388.

Van Doan H, Hoseinifar SH, Sringarm K, Jaturasitha S, Khamlor T, Dawood MA, Esteban MA, Soltani M, Musthafa MS. Effects of elephant's foot (Elephantopus scaber) extract on growth performance, immune response, and disease resistance of nile tilapia (Oreochromis niloticus) fingerlings. Fish Shellfish Immunol. 2019b Oct;93:328-35.

Van Doan H, Hoseinifar SH, Sringarm K, Jaturasitha S, Yuangsoi B, Dawood MAO, Esteban MA, Ringø E, Faggio C. Effects of Assam tea extract on growth, skin mucus, serum immunity and disease resistance of Nile tilapia (Oreochromis niloticus) against Streptococcus agalactiae. Fish Shellfish Immunol. 2019c Oct;93:428-35.

Verma VK, Prakash O, Kumar RSR, Rani KV, Sehgal N. Water hyacinth (Eichhornia crassipes) leaves enhances disease resistance in Channa punctata from Vibrio harveyi infection. J Basic Appl Zool. 2021;82(1):6.

Verma VK, Rani KV, Kumar SR, Prakash O. Leucaena leucocephala pod seed protein as an alternate to animal protein in fish feed and evaluation of its role to fight against infection caused by Vibrio harveyi and Pseudomonas aeruginosa. Fish Shellfish Immunol. 2018 May; 76:324-32.

Wang F, Liu H, Liu F, Chen W. Effects of Chinese yam (Dioscorea oppositifolia L.) dietary supplementation on intestinal microflora, digestive enzyme activity and immunity in rainbow trout (Oncorhynchus mykiss). Aquac Res. 2020;51(11):4698-712.

Wu C, Shan J, Feng J, Wang J, Qin C, Nie G, Ding C. Effects of dietary Radix Rehmanniae Preparata polysaccharides on the growth performance, immune response and disease resistance of Luciobarbus capito. Fish Shellfish Immunol. 2019 Jun;89:641-46.

Wu S. Dietary Astragalus membranaceus polysaccharide ameliorates the growth performance and innate immunity of juvenile crucian carp (Carassius auratus). Int J Biol Macromol. 2020 Apr 15;149:877-81.

Wu Z, Ling F, Song C, Chen W, Wang G. Effects of oral administration of whole plants of Artemisia annua on Ichthyophthirius multifiliis and Aeromonas hydrophila after parasitism by I. multifiliis. Parasitol Res. 2017 Jan; 116(1):91-7.

Xia S-l, Ge X-p, Liu B, Xie J, Miao L-h, Ren M-c, Zhou Q-l, Zhang W-x, Jiang X-j, Chen R-l, Pan L-k. Effects of supplemented dietary curcumin on growth and non-specific immune responses in juvenile wuchang bream (Megalobrama amblycephala). Isr J Aquac - Bamidgeh. 2015 Jan;67.

Xie J, Bo L, Zhou Q, Su Y, He Y, Pan L, Ge X, Xu P. Effects of anthraquinone extract from rhubarb Rheum officinale Bail on the crowding stress response and growth of common carp Cyprinus carpio var. Jian. Aquaculture. 2008 Sep;281:5-11.

https://doi.org/10.17221/96/2023-VETMED

Yang K, Qi X, He M, Song K, Luo F, Qu X, Wang G, Ling F. Dietary supplementation of salidroside increases immune response and disease resistance of crucian carp (Carassius auratus) against Aeromonas hydrophila. Fish Shellfish Immunol. 2020 Nov;106:1-7.

Yao L, Zhang A, Zhang H, Shao J, Wen M, Wang C, Jiang H, Li M. Effects of dietary aqueous extract from Eucommia ulmoides Oliver on growth, muscle composition, amino acid composition and fatty acid composition of rainbow trout (Oncorhynchus mykiss). Pakistan J Zool. 2020;53(2):603-11.

Yousefi M, Ghafarifarsani H, Hoseinifar SH, Rashidian G, Van Doan H. Effects of dietary marjoram, Origanum majorana extract on growth performance, hematological, antioxidant, humoral and mucosal immune responses, and resistance of common carp, Cyprinus carpio against Aeromonas hydrophila. Fish Shellfish Immunol. 2021 Jan;108:127-33.

Yousefi M, Hoseini SM, Vatnikov YA, Kulikov EV, Drukovsky SG. Rosemary leaf powder improved growth performance, immune and antioxidant parameters, and crowding stress responses in common carp (Cyprinus carpio) fingerlings. Aquaculture. 2019 Apr;505:473-80.

Yu Q, Liu M, Xiao H, Wu S, Qin X, Lu Z, Shi D, Li S, Mi H, Wang Y, Su H, Wang T, Li P. The inhibitory activities and antiviral mechanism of Viola philippica aqueous extracts against grouper iridovirus infection in vitro and in vivo. J Fish Dis. 2019 Jun;42(6):859-68.

Zemheri-Navruz F, Acar U, Yılmaz S. Dietary supplementation of olive leaf extract enhances growth performance, digestive enzyme activity and growth related genes expression in common carp Cyprinus carpio. Gen Comp Endocrinol. 2020 Sep;296:113541.

Zhai Q, Li J. Effectiveness of traditional Chinese herbal medicine, San-Huang-San, in combination with enrofloxacin to treat AHPND-causing strain of Vibrio parahaemolyticus infection in Litopenaeus vannamei. Fish Shellfish Immunol. 2019 Apr;87:360-70.

Zhang B, Li C, Wang X, Zhou H, Mai K, He G. The effects of dietary Eucommia ulmoides Oliver on growth, feed utilization, antioxidant activity and immune responses of turbot (Scophthalmus maximus L.). Aquac Nutr. 2019;25(2):367-76.

Zhang H, Ge X, Liu B, Teng T, Zhou Q, Sun C, Song C, Liu B. Comparative transcriptomic and proteomic analysis of the antibacterial activity of emodin on Aeromonas hydrophila. Aquaculture. 2020a Dec;529:735589.

Zhang Q, Xu DH, Klesius PH. Evaluation of an antiparasitic compound extracted from Galla chinensis against fish parasite Ichthyophthirius multifiliis. Vet Parasitol. 2013 Nov;198(1-2):45-53.

Zhang X, Sun Z, Cai J, Wang J, Wang G, Zhu Z, Cao F. Effects of dietary fish meal replacement by fermented moringa (Moringa oleifera Lam.) leaves on growth performance, nonspecific immunity and disease resistance against Aeromonas hydrophila in juvenile gibel carp (Carassius auratus gibelio var. CAS III). Fish Shellfish Immunol. 2020b Jul;102:430-9.

Zhang XP, Li WX, Ai TS, Zou H, Wu SG, Wang GT. The efficacy of four common anthelmintic drugs and traditional Chinese medicinal plant extracts to control Dactylogyrus vastator (Monogenea). Aquaculture. 2014 Jan; 420:302-7.

Zhang Y, Song L, Guo H, Wu J, Wang X, Yao F. Effects of curcumin on growth and liverprotection in common carp, Cyprinus carpio. Pakistan J Zool. 2021 Aug;53(4):1211-20.

Zheng X, Chi C, Xu C, Liu J, Zhang C, Zhang L, Huang Y, He C, He C, Jia X, Liu W. Effects of dietary supplementation with icariin on growth performance, antioxidant capacity and non-specific immunity of Chinese mitten crab (Eriocheir sinensis). Fish Shellfish Immunol. 2019 Jul;90:264-73.

Zhou S, Dong J, Liu Y, Yang Q, Xu N, Yang Y, Gu z, Ai x. Anthelmintic efficacy of 35 herbal medicines against a monogenean parasite, Gyrodactylus kobayashii, infecting goldfish (Carassius auratus). Aquaculture. 2020 May;521:734992.

Zhou S, Li WX, Wang YQ, Zou H, Wu SG, Wang GT. Anthelmintic efficacies of three common disinfectants and extracts of four traditional Chinese medicinal plants against Gyrodactylus kobayashii (Monogenea) in goldfish (Carassius auratus). Aquaculture. 2017 Jan;466:72-7.

Zoral MA, Futami K, Endo M, Maita M, Katagiri T. Anthelmintic activity of Rosmarinus officinalis against Dactylogyrus minutus (Monogenea) infections in Cyprinus carpio. Vet Parasitol. 2017 Nov 30;247:1-6.
